# First SARS-CoV-2 Omicron infection as an effective immune booster among mRNA vaccinated individuals: final results from the first phase of the PRIBIVAC randomised clinical trial

**DOI:** 10.1016/j.ebiom.2024.105275

**Published:** 2024-08-12

**Authors:** Xuan Ying Poh, I. Russel Lee, Chee Wah Tan, Jean-Marc Chavatte, Siew Wai Fong, Yun Shan Goh, Angeline Rouers, Nathan Wong, Anthony Torres-Ruesta, Shirley Y.Y. Mah, Aileen Y.Y. Yeoh, Mihir Gandhi, Nabilah Rahman, Yi Qing Chin, J. Jonathan Lim, Terence J.K. Yoong, Suma Rao, Po Ying Chia, Sean W.X. Ong, Tau Hong Lee, Sapna P. Sadarangani, Ray J.H. Lin, Daniel R.X. Lim, Wanni Chia, Laurent Renia, Ee Chee Ren, Raymond T.P. Lin, David C. Lye, Lin-Fa Wang, Lisa F.P. Ng, Barnaby E. Young

**Affiliations:** a National Centre for Infectious Diseases, Singapore; b Yong Loo Lin School of Medicine, National University of Singapore, Singapore; c National Public Health Laboratory, Singapore; d A∗STAR Infectious Diseases Labs (A∗STAR ID Labs), Agency for Science, Technology and Research (A∗STAR), Singapore, 138648, Singapore; e Emerging Infectious Diseases Programme, Duke-NUS Medical School, Singapore; f Biostatistics, Singapore Clinical Research Institute, Singapore; g Centre for Quantitative Medicine, Duke-NUS Medical School, Singapore; h Saw Swee Hock School of Public Health, Singapore; i Department of Infectious Diseases, Tan Tock Seng Hospital, Singapore; j Lee Kong Chian School of Medicine, Nanyang Technological University, Singapore; k School of Biological Sciences, Nanyang Technological University, Singapore; l Singapore Immunology Network, Singapore

**Keywords:** COVID-19 booster, Hybrid immunity, Cellular response, Humoral immunity, Omicron

## Abstract

**Background:**

Understanding how SARS-CoV-2 breakthrough infections impacts the breadth of immune responses against existing and pre-emergent SARS-CoV-2 strains is needed to develop an evidence-based long-term immunisation strategy.

**Methods:**

We performed a randomised, controlled trial to assess the immunogenicity of homologous (BNT162b2) versus heterologous (mRNA-1273) booster vaccination in 100 BNT162b2-vaccinated infection-naïve individuals enrolled from October 2021. Post hoc analysis was performed to assess the impact of SARS-CoV-2 infection on humoral and cellular immune responses against wild-type SARS-CoV-2 and/or Omicron subvariants.

**Findings:**

93 participants completed the study at day 360. 71% (66/93) of participants reported first SARS-CoV-2 Omicron infection by the end of the study with similar proportions of infections between homologous and heterologous booster groups (72.3% [34/47] vs 69.6% [32/46]; *p* = 0.82). Mean wildtype SARS-CoV-2 anti-S-RBD antibody level was significantly higher in heterologous booster group compared with homologous group at day 180 (14,588 IU/mL; 95% CI, 10,186–20,893 vs 7447 IU/mL; 4646–11,912; *p* = 0.025). Participants who experienced breakthrough infections during the Omicron BA.1/2 wave had significantly higher anti-S-RBD antibody levels against wildtype SARS-CoV-2 and antibody neutralisation against BA.1 and pre-emergent BA.5 compared with infection-naïve participants. Regardless of hybrid immunity status, wildtype SARS-CoV-2 anti-S-RBD antibody level declined significantly after six months post-booster or post-SARS-CoV-2 infection.

**Interpretation:**

Booster vaccination with mRNA-1273 was associated with significantly higher antibody levels compared with BNT162b2. Antibody responses are narrower and decline faster among uninfected, vaccinated individuals. Boosters may be more effective if administered shortly before infection outbreaks and at least six months after last infection or booster.

**Funding:**

Singapore NMRC, 10.13039/100000038USFDA, MRC.

**Research in context**

**Evidence before this study**

We searched PubMed from database inception up to Feb 25, 2024, with no language restrictions, for studies reporting the immunogenicity of mRNA COVID-19 vaccination booster regimens with Omicron breakthrough infections using the search terms “immunogenicity”, “COVID-19 mRNA vaccine booster”, “clinical trial” and “Omicron breakthrough infection”. A cross-sectional cohort study of 58 BNT162b2-boosted healthcare workers found serum neutralizing activity against the Omicron variant to decrease in infection-naïve participants four months post-booster, whereas Omicron breakthrough infection increased surrogate virus neutralisation test (sVNT) inhibition against Omicron within one month post-infection. Omicron sVNT levels were similar regardless of the booster vaccination regimen, whether homologous or heterologous. However, no randomised clinical trials have compared the longevity of hybrid immunity versus vaccine-mediated immunity, and the optimal interval for booster vaccination post-SARS-CoV-2 infection remain unknown.

**Added value of this study**

In this final report of a subject-blinded, randomised-controlled trial of 100 participants, those who experienced breakthrough infections during the Omicron BA.1/2 wave had significantly higher sVNT levels against BA.1 and pre-emergent BA.5 compared with infection-naïve participants. Neutralizing antibodies against BA.1 also waned more rapidly in uninfected than infected participants. Importantly, both hybrid and vaccine-mediated wildtype anti-S antibody levels declined significantly six months post-infection or post-booster. Our data support the administration of additional booster doses at least six months after the last SARS-CoV-2 infection or vaccine dose to maximize the efficacy and protection of COVID-19 vaccination programs.

**Implications of all the available evidence**

These data are of paramount importance to inform public health measures on the optimal interval for booster vaccination in a highly vaccinated population with high incidence of Omicron infections.

## Introduction

Severe acute respiratory syndrome coronavirus 2 (SARS-CoV-2) vaccination is highly effective at reducing the risk of severe disease and mortality from coronavirus disease 2019 (COVID-19). In Singapore, the first two COVID-19 vaccines to be granted authorization by the Health Sciences Authority (HSA) were the mRNA vaccines BNT162b2 and mRNA-1273 developed by Pfizer-BioNTech and Moderna, respectively.^1^^,^^2^ 95% of adults in Singapore received one of these vaccines as part of their primary vaccination series.^3^

Despite the effectiveness of BNT162b2 and mRNA-1273 at preventing complications of COVID-19, vaccination does not result in sterilizing immunity. This is due to their limited induction of mucosal immunity, the emergence of immune evasive SARS-CoV-2 variants,^4^^,^^5^ and waning antibody levels after vaccination.^6^ As a result, booster vaccinations are necessary, and there is a need to develop an evidence-based long-term immunisation strategy. Key considerations include who should be prioritized for boosting, the optimal interval for booster vaccination as well as the effect of mixing vaccine types (for example, homologous booster [same as the primary vaccine] versus heterologous booster [different from the primary vaccine]).

Recommendations for vaccine boosters also need to factor in the impact of prior SARS-CoV-2 infection on immune responses and vaccine effectiveness. The low neutralisation titres against Omicron following a two-dose primary vaccination series, although boosted by a third ancestral vaccine dose,^7^^,^^8^ remain significantly lower than the neutralisation titres against ancestral and Delta strain.^9^ Recent evidence has highlighted the benefits of SARS-CoV-2 infection in protecting against re-infection and severe disease.^10^^,^^11^ However, convalescent sera demonstrate substantially reduced neutralisation titres against Omicron compared with both the ancestral strain and the homologous strain causing infection, suggesting that previously infected individuals may have little protection against Omicron re-infection.^9^ Due to strict containment strategies from 2020-21, only 3.5% of the population in Singapore was recorded to be SARS-CoV-2 infected—and most infections were due to the Delta variant.^12^^,^^13^ This provided a unique opportunity to study the effects of Omicron infection in a naïve but vaccinated population in 2022.^14^^,^^15^

PRIBIVAC is a randomised, subject-blinded phase 4 trial designed to investigate the immunogenicity, safety and reactogenicity of BNT162b2 versus mRNA-1273 booster vaccination (third dose) in individuals who had received the BNT162b2 primary series at least 6 months prior to enrolment.^16^ While not intended to study SARS-CoV-2 infections when the trial was designed, the follow-up phase of this trial coincided with the emergence of Omicron, and a large community outbreak in Singapore.^12^^,^^17^ Interim results of phase A (up to day 28 post enrolment) were reported previously.^18^ Here, we report the final set of results for phase A up until the last follow-up visit on day 360.

## Methods

### Enrolment, randomisation and clinical procedures

We enrolled 100 adult volunteers in Phase A of the PRIBIVAC trial between October and November 2021 at the National Centre for Infectious Diseases, Singapore. Study participants were randomised 1:1 to receive one intramuscular dose of either BNT162b2 30 μg (0.3 mL) or mRNA-1273 50 μg (0.25 mL).

All study participants received BNT162b2 as their primary vaccination series at least six months prior to study enrolment. Key exclusion criteria included a known history of SARS-CoV-1 or SARS-CoV-2 infection, recipient of SARS-CoV-2 monoclonal antibody therapy, and presence of immune compromise or on immunosuppressants. Sex was self-reported by study participants. Full entry criteria are available in the published PRIBIVAC trial protocol and ClinicalTrials.gov (NCT05142319).^16^

Randomisation was stratified by age (<60 years, ≥60 years) and time from the second vaccine dose administered (6–9 months, >9 months). The study team from the Singapore Infectious Disease Clinical Research Network randomised study participants using a web-based randomisation system hosted by the Singapore Clinical Research Institute. The randomisation list with randomised permuted block size of 4 was generated by the trial statistician. Block size was revealed only after study recruitment had completed. Study participants are blinded to the vaccine allocation up to day 28 post-booster to reduce the risk of bias in participant-reported AEs. Primary outcome assessors and laboratory personnel performing sample analysis were masked from the study allocation of participants.

Blood samples were collected pre-booster (day −28 to day 0) and post-booster day 7 (±2 days), day 28 (±7 days), day 180 (±14 days) and day 360 (±14 days) for various immunogenicity assays. Participants were given a diary to record solicited and unsolicited local and general symptoms experienced in the first 7 days after the booster vaccination. Surveillance for COVID-19 infection was performed by measuring anti-nucleocapsid (anti-N) antibody levels and by asking participants to report positive antigen rapid test (ART) and/or PCR results throughout the study period.

The primary objective for phase A was to determine whether heterologous vaccine booster (i.e., mRNA-1273) led to a non-inferior humoral immune response against wild-type SARS-CoV-2 and VOC at day 28 compared with homologous vaccine booster (i.e., BNT162b2). Key secondary endpoints included measurements of the humoral immune responses at days 180 and 360, SARS-CoV-2-specfic T and B cell responses at days 28, 180 and 360, and surveillance for COVID-19 infection throughout the study period.

### Quantitative and qualitative measurements of SARS-CoV-2 anti-S-RBD and anti-N immunoglobulins

Wildtype SARS-CoV-2 anti-N and anti-spike receptor-binding domain (anti-S-RBD) immunoglobulins levels were measured using Elecsys® (Roche, Basel, Switzerland) chemiluminescent immunoassays according to the manufacturer's instructions. Antibody titres in U/mL from the Elecsys® anti-S-RBD assays are equivalent to the World Health Organization's standard binding antibody units per millilitre,^19^ and no conversion was performed. Elecsys® anti-N results were interpreted as follows: cut-off index (COI) <1.0, negative; COI ≥1.0, positive.

### Surrogate virus neutralisation test (sVNT)

SARS-CoV-2 neutralizing antibodies were measured with a multiplex sVNT assay which detects total immunodominant neutralizing antibodies targeting the viral spike RBD in an isotype- and species-independent manner.^20^^,^^21^ Briefly, AviTag-biotinylated RBD proteins from wildtype SARS-CoV-2 and other VOCs were coated on a MagPlex Avidin microsphere (Luminex) at 5 μg/1 million beads. RBD-coated microspheres (600 beads/antigen) were pre-incubated with serum at a final concentration of 1:20 or greater for 15 min at 37 °C with 250 rpm agitation. 50 μL phycoerythrin-conjugated human angiotensin-converting enzyme 2 (GenScript 2 μg/mL) was then added to the well and incubated for 15 min at 37 °C with agitation, followed by two phosphate buffered saline (PBS)-1% bovine serum albumin washes. The final readings were acquired on the MAGPIX system. Results were interpreted with reference to the nominal “seronegative” threshold of 30%.^20^

### Wildtype SARS-CoV-2 full-length spike protein assay

Spike-specific antibodies were measured by Spike protein flow cytometry-based assay.^22^ Briefly, cells expressing the wildtype spike protein were seeded at 1.5 × 10^5^ cells per well in 96-well V-bottom plates (Thermo Fisher Scientific). Cells were incubated with human plasma samples (diluted 1:100 in 10% fetal bovine serum [FBS]; HyClone) for 30 min at 4 °C, followed by a secondary 30 min incubation at 4 °C with double stain comprising Alexa Fluor 647-conjugated anti-human IgG (1:500 dilution; ThermoFisher Scientific) and propidium iodide (1:2500 dilution; Sigma–Aldrich). Cells were acquired using a BD Biosciences (Becton–Dickinson) LSR4 laser and analysed using FlowJo (Tree Star, BD Biosciences). The assay was performed as two independent experiments.

### Quantitative measurement of SARS-CoV-2-specific memory B cell (MBC) and T cell responses

High-dimensional flow cytometric detection of SARS-CoV-2 T cell responses and quantification of SARS-CoV-2-specific MBCs by B-cell ELISpot assay was performed as previously described.^23^^,^^24^

### Sample size calculation

A mean SARS-CoV-2 sVNT inhibition level of 84% (standard deviation (SD), 15%) at 28 days after administration of the second vaccine dose was observed in a local study.^8^ A common SD was used in the two treatment groups. We predicted that immunogenicity in the homologous arm will be boosted back to approximately the same level after administration of the third dose. With a margin of −10%, a sample size of 87 participants per arm was calculated to be needed to conclude non-inferiority in the heterologous arm relative to homologous arm with 80% power. The inferiority margin of 10% was selected to preserve at least 80% of the treatment effect in the standard arm. The sample size is calculated at a one-sided 2.5% significance level and accounts for an attrition rate of 15%.

### Statistics

Data were assessed for normal distribution by visual inspection. Non-parametic tests were conducted when the assumption of normality was violated. Categorical variables were analysed as absolute numbers and relative frequencies and compared using chi-square test or Fisher's exact test when the value in any of the cells is below five. Continuous variables were analysed as median and interquartile range (IQR) or mean and SD and compared using Mann–Whitney U test or independent *t* test as appropriate based on the results of assessing normality using GraphPad Prism version 10.2.3. Homogeneity of variance was assessed using F test prior to conducting the independent *t* test. Student's *t* test was used when equal variance was assumed, and Welch's *t* test for unequal variance. One-way ANOVA with Tukey's multiple comparisons test or Kruskal–Wallis test with Dunn's multiple comparisons test was used when comparing 3 or more groups. Anti-S-RBD antibody titres were log_10_-transformed for all statistical analysis.

For the post-hoc analysis, we employed multiple linear regression to explore the effect of booster vaccine (mRNA-1273 or BNT162b2) on post-vaccination anti-S-RBD antibody titres while controlling for baseline values. A multiple linear regression model of post-vaccination anti-S-RBD antibody titres was constructed that included booster vaccine group, randomization stratification factors [age (<60 years; ≥60 years) and time since vaccination (6–9 months; >9 months)], sex, charlson comorbidity index (0; ≥1) and COVID-19 infection status. Normality and homogeneity of variance of residuals were assessed using graphical methods via QQ plot (actual residuals on x-axis vs predicted residuals on y-axis) and residual plot (predicted value on x-axis vs residuals on y-axis) respectively.

Cumulative incidence of COVID-19 infections and hazard ratio (HR) between vaccine arms were analysed by Log-rank test. The analysis started from the date of booster vaccination (Day 0) and ended when a participant had COVID-19 infection or withdrew or died or the end of the study was reached (in the latter three circumstances, the participant data were considered to have been censored). Participants who did not receive the booster vaccination were excluded from the analysis. By the end of the study, 32% and 33% of participant data were censored in homologous arm (4% withdrew, 2% died and 26% remained COVID-19 naïve by the end of study) and heterologous arm (4% withdrew and 29% remained COVID-19 naïve by the end of study) respectively.

Paired MBC levels in each infection group were analysed by Wilcoxon match paired test.

The data were analysed for all outcomes in a modified intention-to-treat (mITT) population, which included all randomly assigned participants except those who never received the booster vaccination, withdrew from the study, died or had a missing blood sample at day 360. Statistical significance was defined as *p* < 0.05. Statistical analyses were performed using GraphPad Prism version 10.2.3.

### Ethics statement

This study received ethical approval from the Institutional Review Board: National Healthcare Group Domain Specific Review Board (NHG DSRB, study reference 2021/00821). All participants provided written informed consent to participate.

### Role of the funding source

The funders of the study had no role in study design, data collection, data analysis, data interpretation, or writing of this report.

## Results

### Participants

**Fig. 1:**
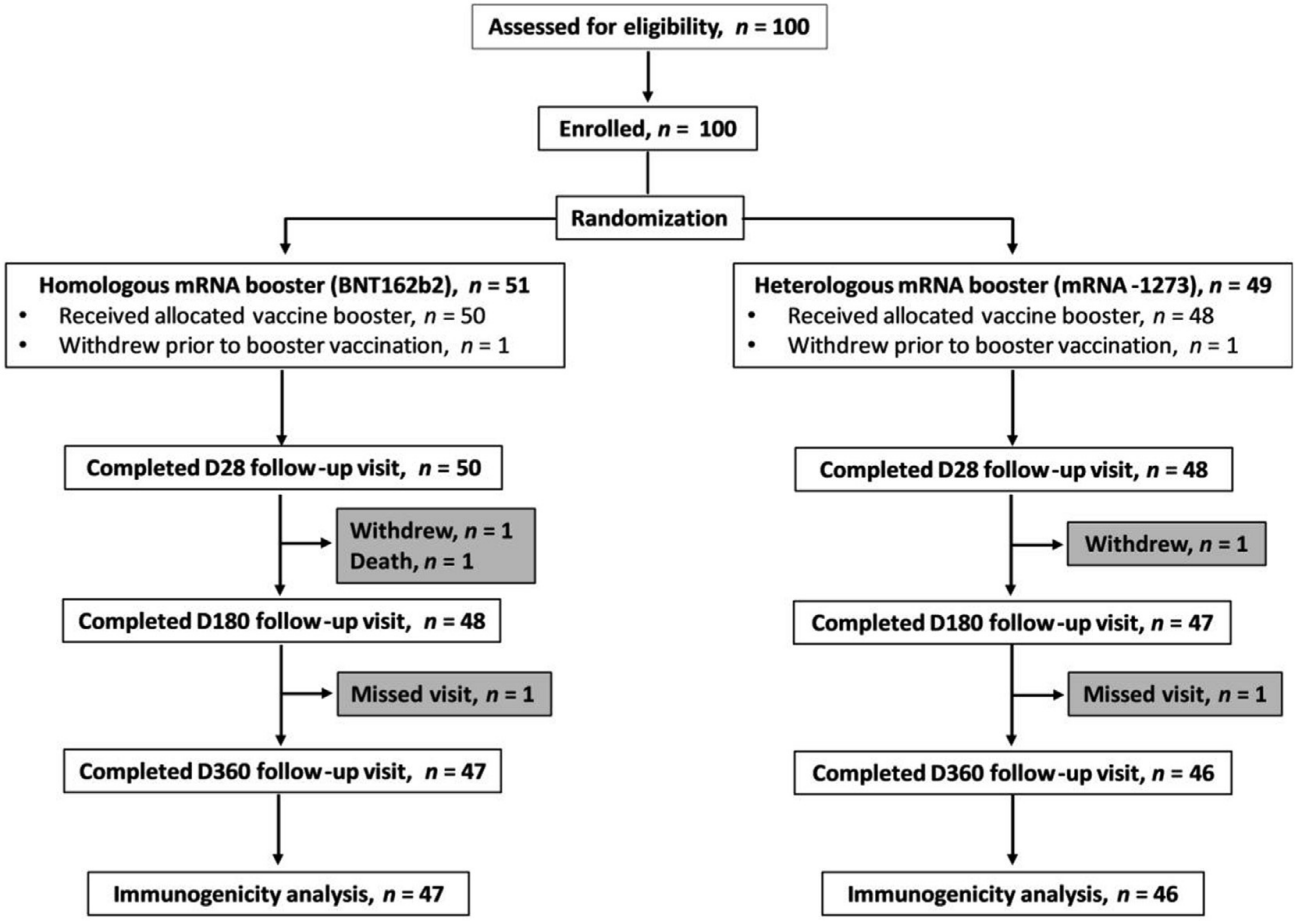
Consort diagram depicting the disposition of trial participants, from enrolment to exposure grouping to flow through study analysis.

51 participants were randomised to receive a homologous BNT162b2 booster and 49 to a heterologous mRNA-1273 booster. One participant from each study arm withdrew prior to the booster vaccination visit, one from each arm withdrew prior to the day 180 visit, one from the homologous arm died prior to the day 180 visit (unrelated to study treatment), and one from each arm missed the day 360 visit. This resulted in a complete dataset of *n* = 47 in the homologous arm and *n* = 46 in the heterologous arm (Fig. 1). Demographics and baseline characteristics of the final cohort (*n* = 93) summarized by vaccine booster type are shown in Table 1. The safety and reactogenicity outcomes had been reported in the interim analysis previously.^18^

**Table 1:** Demographics and baseline characteristics of the final cohort summarised by type of vaccine booster received.

	BNT162b2 booster vaccination (*n* = 47)	mRNA-1273 booster vaccination (*n* = 46)
Age in years, median (IQR)	54 (31–69)	60 (35–65)
Male, no. (%)	20 (42.6)	20 (43.5)
Chinese, no. (%)	42 (89.4)	43 (93.5)
Charlson comorbidity index, median (IQR)	0 (0–0)	0 (0–0)
Days since second vaccine dose, median (IQR)	240 (209–264)	235 (202–256)
Former smoker, no. (%)	1	1

**Fig. 2:**
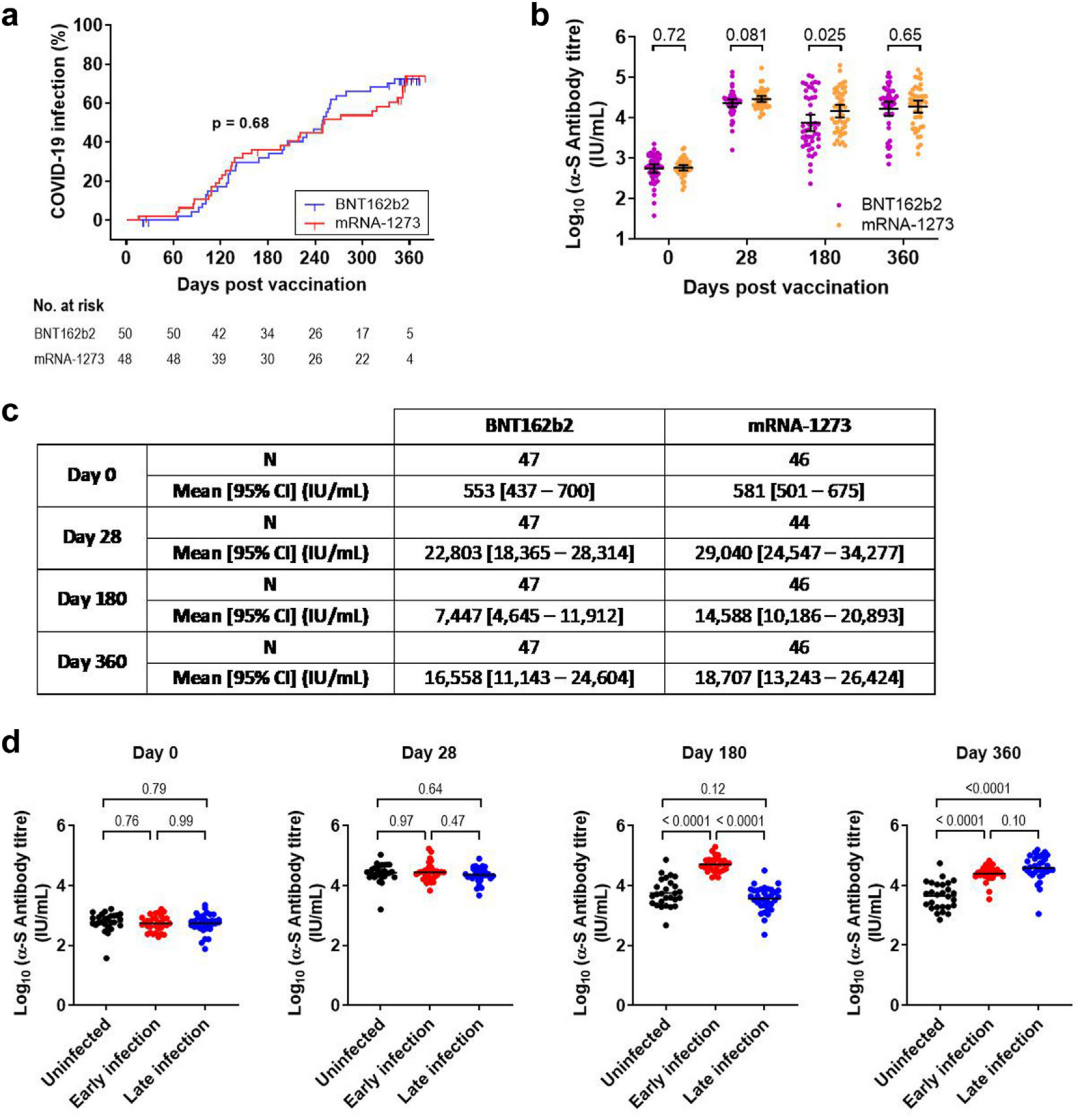
Cumulative incidence of COVID-19 infection and longitudinal measurements of SARS-CoV-2 anti-S-RBD titres over a one-year study period. **(a)** Cumulative incidence of COVID-19 infection in participants who received the homologous BNT162b2 (*n* = 47) and heterologous mRNA-1273 (*n* = 46) booster vaccines. COVID-19 infection was assessed by participants' self-performed COVID-19 Antigen Rapid Test (ART) and/or SARS-CoV-2 anti-nucleocapsid (N) seroconversion. The censored patient is denoted by a downward blip. Data was analysed by Log-rank test. **(b)** Level of anti-S-RBD antibody between homologous (BNT162b2) and heterologous (mRNA-1273) vaccine arms. Data was analysed using independent t test. Graph depicts mean and 95% CI. **(c)** Summary data. **(d)** Level of anti-S-RBD antibody between infection groups. Bar represents mean. Data was analysed using one-way ANOVA with Tukey's multiple comparison test.

The median duration of follow-up after the booster dose was 350 days (IQR 348–356) in homologous arm and 351 days (IQR 348–358) in heterologous arm. All study participants were COVID-19 naïve at enrolment and there were no COVID-19 infections in the first 28 days post boost. 71% (66/93) of the cohort reported infections by the end of the study, of which 34.4% (32/93) occurred from days 29–180 (early infection group) and 36.6% (34/93) from days 181–360 (late infection group). The proportion of infections in the homologous vaccinated arm was similar to the heterologous arm (34/47 [72.3%] vs 32/46 [69.6%], *p* = 0.77, Chi-square). Compared to homologous arm, heterologous vaccinated participants were observed to have a similar risk of COVID-19 infection (HR = 0.90, 95% CI, 0.56–1.46, *p* = 0.68, Log-rank test). Time from booster vaccination to COVID-19 infection was also similar (*p* = 0.68, Log-rank test; Fig. 2a). Demographics and baseline characteristics of the cohort (*n* = 93) summarized by infection status/period (uninfected, early infection, late infection groups) are shown in Table 2.

**Table 2:** Demographics and baseline characteristics of the final cohort summarised by infection status/period.

(COVID-19 infection period)	Uninfected	Early infection (Between days 29 and 180)	Late infection (Between days 181 and 360)	*p*-value
N	27 (29.0%)	32 (34.4%)	34 (36.6%)	
Age in years, median (IQR)	48 (31–67)	51 (31–67)	62 (39–65)	0.61
Male, no. (%)	12 (44.4)	13 (40.6)	16 (47.1)	0.87
Chinese, no. (%)	24 (88.9)	29 (90.6)	32 (94.1)	0.81
Homologous BNT162b2 booster, no. (%)	13 (48.1)	15 (46.9)	19 (55.9)	0.73
Charlson comorbidity index, median (IQR)	0 (0–0)	0 (0–0)	0 (0–0)	0.90
Days since second vaccine dose, median (IQR)	250 (206–266)	239 (206–259)	221 (205–255)	0.35
Former smoker	0 (0%)	1 (3.1%)	1 (2.9%)	1.0
Anti-S RBD titre, mean (95% CI) (IU/mL)				
Day 0	618 (457–834)	545 (442–671)	551 (430–706)	0.74
Day 28	26,730 (19,907–35,810)	27,733 (21,727–35,400)	22,856 (18,578–28,119)	0.47
Day 180	5728 (3767–8730)	51,286 (42,073–62,661)	3698 (2673–5117)	<0.0001
Day 360	4426 (2931–6683)	24,660 (19,999–30,339)	38,282 (27,353–53,703)	<0.0001

### Anti-spike antibody levels against wildtype SARS-CoV-2

Mean wildtype SARS-CoV-2 anti-S- RBD antibody levels between intervention groups were similar at all time points, except at day 180 post-booster where the heterologous booster group had a significantly higher level compared with the homologous group (14,588 IU/mL; 95% CI, 10,186–20,893 vs 7447 IU/mL; 4646–11,912; *p* = 0.025, Student's *t* test) (Fig. 2b and c). As 34.4% of participants experienced breakthrough infections between days 29–180, a boost in anti-S-RBD antibody titres was expected and we adjusted for COVID-19 infection as well as stratification factors in a multiple linear regression model. On multiple regression, anti-S-RBD titres at day 180 were significantly higher with heterologous booster vaccination (*p* = 0.0040) and COVID-19 infection (*p* < 0.0001).

A similar finding was observed when analysing only the participants who were COVID-19 naïve up to day 180 (*n* = 61), by anti-S-RBD (Supplemental Fig. S1); full-length spike (Supplemental Fig. S2) and antibody neutralisation (Supplemental Fig. S3), with levels against wildtype SARS-CoV-2 significantly higher among individuals who received the heterologous mRNA-1273 booster compared with homologous BNT162b2 booster. By day 360 when there were only 27 participants left uninfected (13 homologous; 14 heterologous), we could not detect a difference in the levels of anti-S-RBD or neutralizing antibodies between the heterologous and homologous groups (Supplemental Fig. S4).

Infection boosted anti-S-RBD levels to above the Day 28 peak in both early and late infection groups: early infection group's mean anti-S-RBD level 51,286 IU/mL; 95% CI, 42,073–62,661, fold increase D180/28 1.8 (95% CI 1.4-fold–2.4-fold; *p* = 0.0008, RM one-way ANOVA with Tukey's multiple comparison test); late infection group's mean anti-S-RBD level 38,282 IU/mL; 95% CI, 27,353–53,703, fold increase D360/28 1.6 (95% CI 1.2-fold–2.2-fold; *p* = 0.034, RM one-way ANOVA with Tukey's multiple comparison test) (Fig. 2d and Supplemental Fig. S5).

Analysis of full-length spike-specific antibodies (Supplemental Fig. S6) and by sVNT (Supplemental Fig. S7) demonstrated the same trend as RBD-specific antibodies.

### Antibody neutralisation against Omicron BA.1, BA.2 and BA.5

**Fig. 3:**
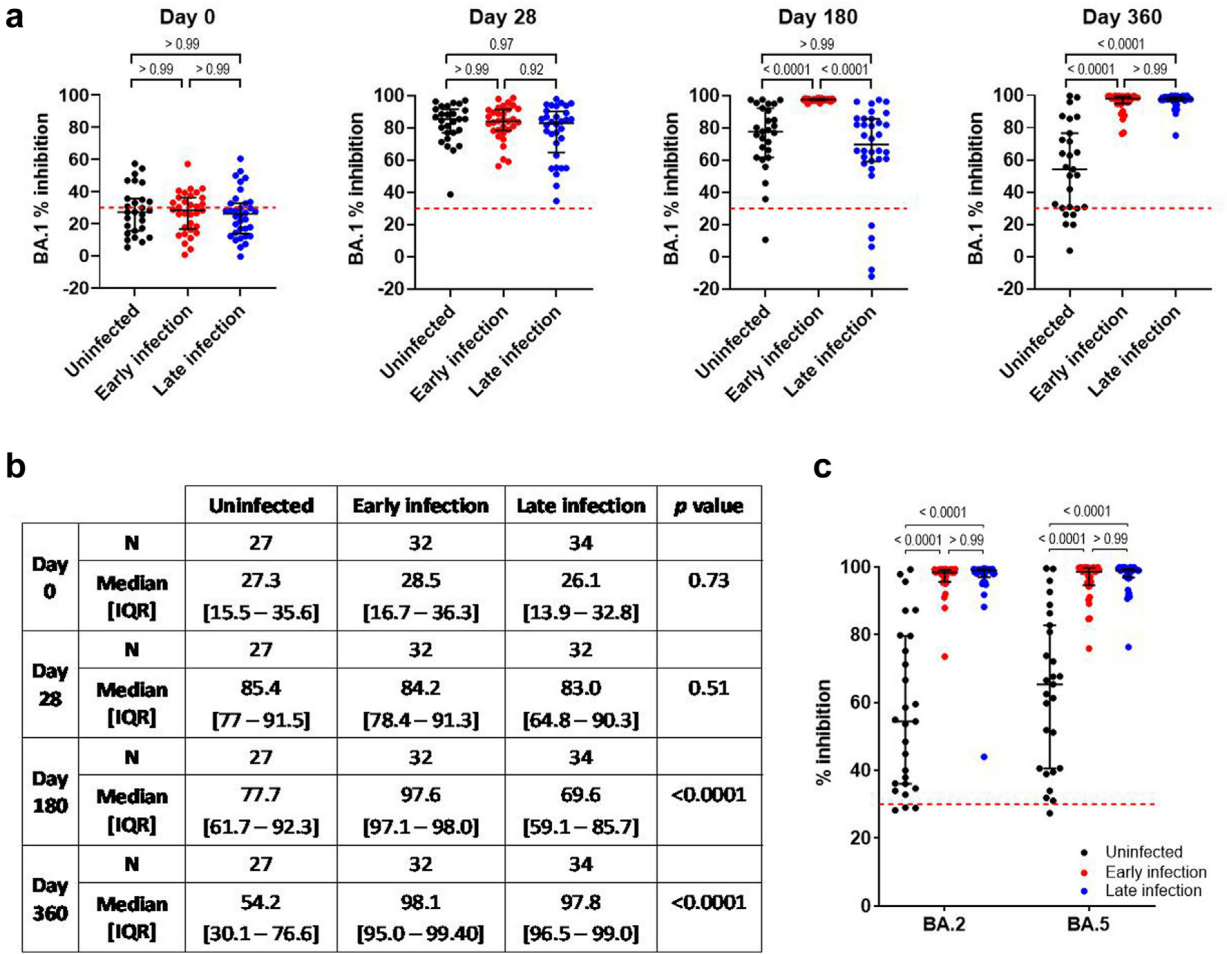
Longitudinal measurements of antibody neutralisation against Omicron BA.1 over a one-year study period to examine infection-induced humoral immune response. **(a)** BA.1 sVNT inhibition level between infection groups. **(b)** Summary data. **(c)** Level of neutralizing antibodies against Omicron BA.2 and BA.5 at day 360 post-booster. Data was analysed using Kruskal–Wallis test with post-hoc Dunn's multiple comparisons test. Red dotted line indicate inhibition of 30% (nominal “seronegative” threshold). Bars represent median and IQR.

As COVID-19 infection in trial participants occurred during the Omicron wave with BA.1/2, we measured the inhibition level against Omicron BA.1, BA.2 and pre-emergent BA.5 via sVNT. Although most participants (61.3%) were seronegative for BA.1 pre-booster, a booster vaccine dose significantly boosted the level of neutralizing antibodies against BA.1 by day 28 post-booster and were similar between vaccine groups at all time points (Supplemental Fig. S8). Between infection groups, the same trend was observed as anti-S-RBD antibodies. Importantly, natural COVID-19 infection significantly boosted the inhibition level against not just the likely infecting Omicron variant (BA.1/2) but also against pre-emergent BA.5 in both early and late infection groups (Fig. 3). Without COVID-19 infection as an immune booster, neutralizing antibodies against BA.1 in participants who were uninfected waned to a median of 54.2% (IQR 30.1%–76.6%) by day 360 post-booster (Fig. 3a and b), whereas wildtype SARS-CoV-2 inhibition level remained high at median 95.1% (IQR 86.6%–96.4%) (Supplemental Fig. S7a).

### T and B cell response against wildtype SARS-CoV-2

**Fig. 4:**
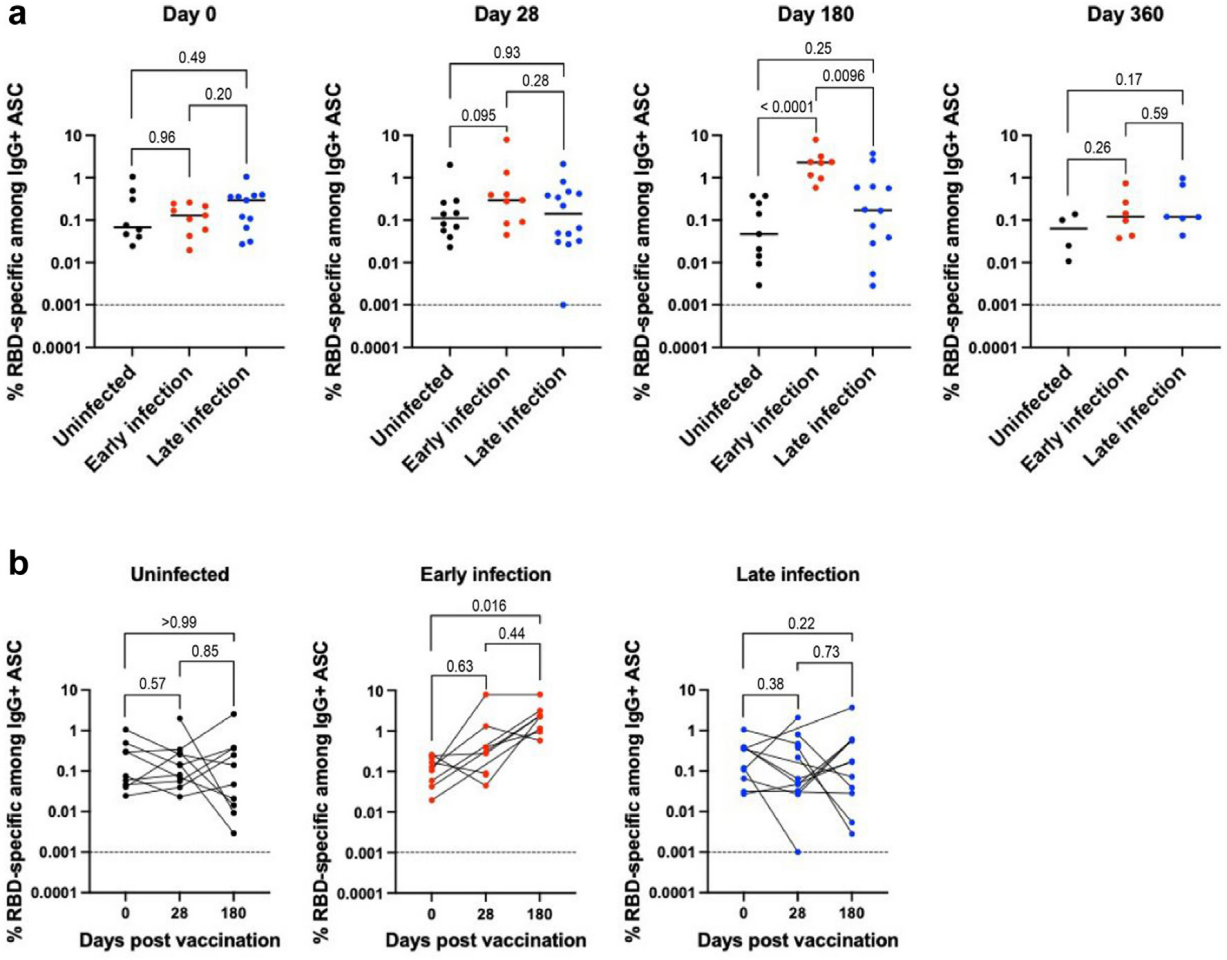
Longitudinal measurements of memory B cells against wildtype SARS-CoV-2 receptor binding domain over a one-year study period to examine vaccine- and infection-induced humoral immune response. Participants (*n = 39*) had the BNT162b2 primary vaccination series prior to enrolment, and received either BNT162b2 or mRNA-1273 booster at study day 1. Within the cohort, 33% (13/39) had early COVID-19 infections and 41% (15/39) had late COVID-19 infections. Data was analysed using Mann–Whitney U (a) or Wilcoxon match paired (b) test. Each dot represents data from one participant. **(a)** Data grouped by study visits (day 0 [pre-booster], post-booster days 28, 180 and 360). **(b)** Data grouped by infection status/period (uninfected, early infection, late infection).

SARS-CoV-2-specific B cell responses against wildtype SARS-CoV-2 RBD were analysed for a subset of the cohort (*n* = 39) with sufficient PBMCs available. At day 180, early infection group had significantly higher MBC levels compared with uninfected (*p* < 0.0001, Mann–Whitney U test) or late infection group (*p* = 0.0096, Mann–Whitney U test) (Fig. 4a). At day 360, there was a trend towards higher MBC levels in early and late infection groups compared with those who were uninfected, although this did not achieve statistical significance (*p* = 0.26 and *p* = 0.17 respectively, Mann–Whitney U test). While COVID-19 infection resulted in higher MBC levels, booster vaccination did not lead to a significant increase in MBC levels (Fig. 4b).

**Fig. 5:**
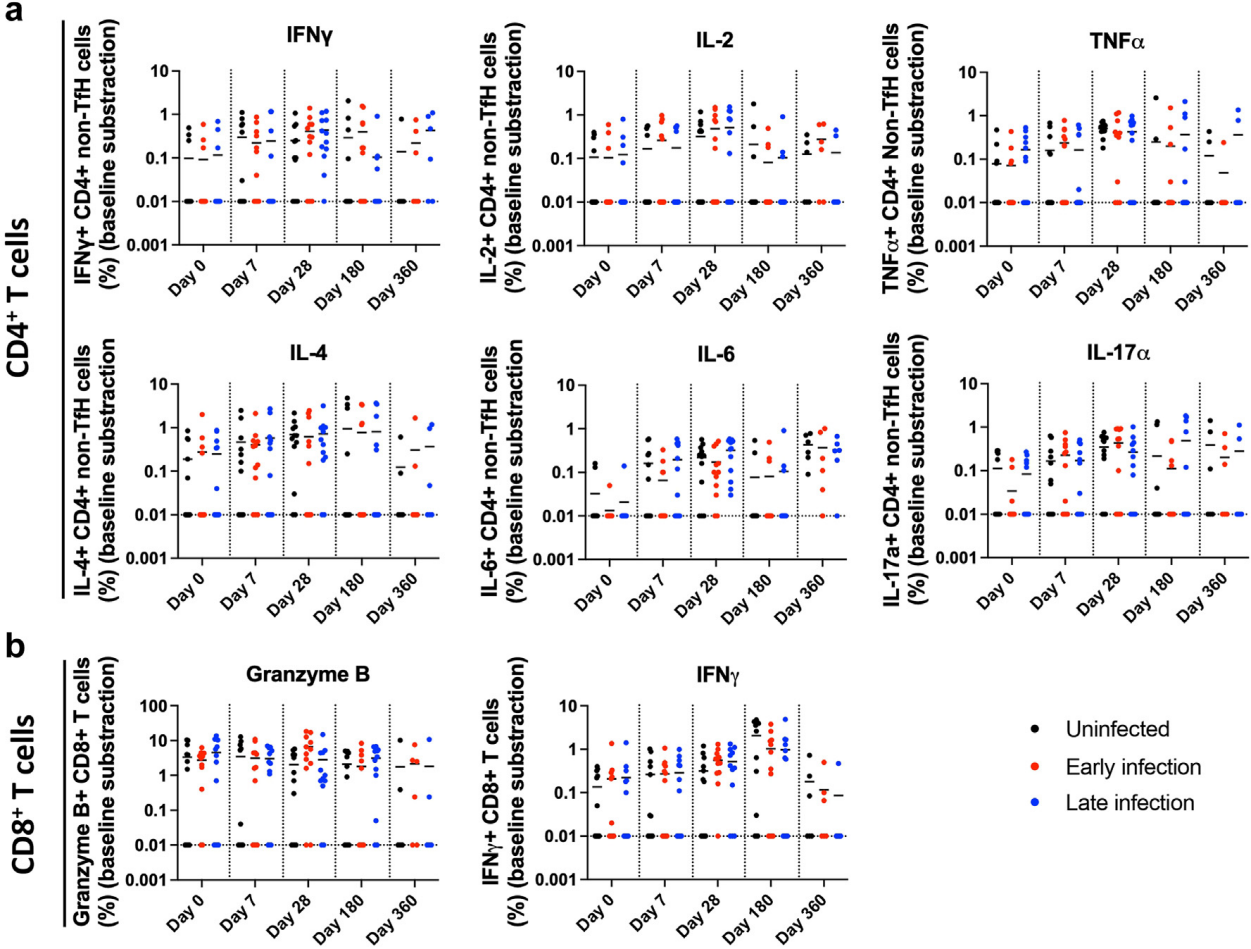
Longitudinal measurements of cytokine-expressing T cell subsets against wildtype SARS-CoV-2 peptides over a six-month study period to examine vaccine- and infection-induced cellular immune responses. Participants (*n = 36*) had the BNT162b2 primary vaccination series prior to enrolment and received either BNT162b2 or mRNA-1273 booster at study day 1. Within the cohort, 33% (12/36) had early COVID-19 infections and 33% (12/36) had late COVID-19 infections. Data was analysed using Krustal–Wallis test with Dunn's multiple comparisons test. *p*-values for pairwise comparison between infection groups at each time point and cytokine is shown in Supplemental Table S2. Each dot represents data from one participant. Cytokines expressed by **(a)** CD4^+^ T cells and **(b)** CD8^+^ T cells.

Circulating virus-specific T cell responses against pooled peptides spanning the entire wildtype SARS-CoV-2 spike protein were also measured for a subset of the cohort (*n* = 36). In general, booster vaccination increased the overall intracellular expression of CD4^+^ and CD8^+^ T cell cytokines at days 28, 180 and 360 compared with pre-booster levels at day 0 (Fig. 5). Unlike what was observed in the humoral responses, COVID-19 infection (regardless early or late) had negligible effect on the expression patterns of CD4^+^ and CD8^+^ T cell subsets as the cytokines they produced remain at comparable levels compared with participants who were uninfected at the various study time points.

## Discussion

COVID-19 mRNA vaccination generates protective immunity against SARS-CoV-2 by inducing robust antibody responses as well as memory B and T cells that are rapidly activated upon antigen exposure. Although phase A of the trial was designed to compare the immune response induced by heterologous versus homologous mRNA booster vaccines (third dose), the Omicron wave in 2022 resulted in 71% of trial participants experiencing breakthrough first SARS-CoV-2 infections from day 29 post boost up until the end of study at day 360. Consequently, we performed post hoc analysis to assess the impact of SARS-CoV-2 infection on humoral and cellular immune responses. It is becoming increasingly apparent that both infection-acquired and vaccine-induced immunity are beneficial in protecting against severe disease and death.^10^

The longitudinal nature of this study enabled a detailed analysis of the longevity of immunity over time through three (3-dose mRNA vaccinations) or four (3-dose mRNA vaccinations plus Omicron breakthrough infection) antigen exposures and are useful data to inform future long-term vaccination strategy. At day 180, the higher antibody titres against wildtype SARS-CoV-2 in participants who received mRNA-1273 booster may be due to the higher antigenic dosage (50 mcg) as compared with BNT162b2 (30 mcg), as dose-escalation trials of mRNA COVID-19 vaccines have shown a dose-dependent antibody response.^25^, ^26^, ^27^ Without natural infection as an immune booster, anti-S-RBD titres against wildtype SARS-CoV-2 declined from peak levels (at one month post booster vaccination) but remained significantly above pre-boost levels even at one year post boost. The antibody titres stabilised between 6 and 12 months post boost, which is in keeping with ongoing antibody production from long-lived plasma cells in the later phases of immune memory after vaccination.^28^ However, the rapid decay of neutralizing antibodies against BA.1 and low neutralisation against BA.5 in participants without breakthrough infection suggest that a single booster vaccine dose does not provide sufficient protection against Omicron subvariants. Additional booster doses are likely to be important for this population to enhance the neutralizing breadth of antibodies against emerging VOCs. Timing boosters close to surges of infections may improve their effectiveness, and is a strategy that is supported by the rapid immune response to mRNA vaccines as compared with traditional inactivated vaccine designs—where for example waning of protection against influenza infections over the course of a season has been observed.^29^^,^^30^

Participants who experienced breakthrough infections had significantly increased anti-S-RBD antibody levels post-infection against wildtype SARS-CoV-2 and antibody neutralisation against BA.1 relative to one month post boost (third antigen exposure). Further, among individuals who were likely to have been infected by BA.1/2, antibody levels were also significantly higher against BA.5. This broadened immune response induced by a combination of pre-Omicron (here referring to the ancestral mRNA booster vaccination) and Omicron immunity (breakthrough infection) suggests that these individuals would have been better protected during the subsequent BA.5 wave compared with infection-naïve individuals.^31^ Indeed, local epidemiological data found BA.1/2-infected and boosted individuals to have a 2.5–4.5x lower incidence of medically attended symptomatic BA.4/5 reinfection than infection-naïve boosted individuals.^32^ The enhanced immune response following an Omicron infection concurs with other studies investigating the impact of vaccination or SARS-CoV-2 infection history on Omicron-induced immune boosting,^33^^,^^34^ and aligns with epidemiological evidence on immune imprinting.^35^^,^^36^ Collectively, our data indicates that when scheduling boosters both the time since last COVID-19 infection and last vaccine dose should be considered. This is an area that requires further study particularly for interpreting observational studies of vaccine effectiveness against symptomatic illness (as opposed to severe disease).^37^

Like antibody responses, breakthrough infections enhanced the level of circulating MBC. The restoration of waning humoral response due to natural infection might extend the longevity and defer the necessity for booster vaccinations, particularly in healthy individuals with “hybrid” immunity. We also characterised wild-type spike-specific T cell responses and observed that booster vaccination led to an elevation in virus-specific CD4^+^ T cells producing Th1, Th2 and Th17 cytokines and virus-specific CD8^+^ T cell responses. Similar to a prospective cohort study conducted in Sweden,^38^ Omicron breakthrough infections enhanced wild-type spike-specific T cell responses but it did not achieve statistical significance. Nevertheless, they may potentially augment non-spike-specific T cell responses as natural infection is known to induce broader T cell responses—recognizing antigens found in the membrane and nucleoprotein.^38^^,^^39^

There are several limitations to the study. Although we investigated the immunological responses of a third dose of COVID-19 mRNA vaccine, the study was not powered to examine vaccine efficacy, clinical outcomes, and the correlates of protection remain to be defined. Secondly, enrolment to Phase A of the study was terminated prematurely due to escalation of community booster vaccination program. As such, the sample size required to test non-inferiority of heterologous versus homologous booster was not achieved and results are presented descriptively. The lack of association between antibody titres and the occurrence of breakthrough infections may also be due to the relatively small sample size, which lacks the statistical precision needed to detect effects. Further studies in large population cohorts with routine monitoring of COVID-19 infections and mucosal immunity are required to better define the immune correlates of protection. Furthermore, due to limited blood samples and insufficient number of PBMCs isolated, only a subset of the cohort was analysed for B and T cell responses, and Omicron-specific cellular responses were not assessed. Lastly, this study recruited predominantly healthy individuals and therefore the immunological responses observed may not be representative of immunocompromised individuals who are at higher risk of developing severe COVID-19.

Overall, the results from this clinical trial supports the utility of booster vaccines to recall immunological memory and boost circulating antibody levels, not only to the ancestral SARS-CoV-2 strain but also against antigenically advanced strains. Phase D is currently ongoing to investigate how a fourth dose boosting with an Omicron-specific vaccine [Comirnaty Bivalent (Original/Omicron BA.4/5) or Moderna/Spikevax Bivalent (Original/Omicron BA.1)] will enhance recall responses compared with boosting with the ancestral strain. This information may help inform public health policy makers on the appropriate time for booster vaccination in vaccinated infection-naïve individuals versus individuals with a “hybrid” immunity.

## Contributors

*Concept and design:* BY, LN, LFW, DCL, RTPL, ECR, LR, MG, NR.

*Patient recruitment and data collection:* BY, DCL, SR, PYC, SO, THL, SS, YQC, JJL, TY.

*Analysis and interpretation of data:* XYP, IRL, CWT, JMC, SWF, YSG, AR, NW, ATR, SM, AY, MG, NR, DL, WC, LR, RTPL, DCL, LFW, LN, BY.

*Drafting of the manuscript:* XYP, IRL, SWF, YSG, AR, NW, ATR, BY.

*Critical revision of the manuscript for important intellectual content:* XYP, IRL, CWT, JMC, SWF, YSG, AR, NW, ATR, SM, MG, NR, AY, YQC, JJL, TY, SR, PYC, SO, THL, SS, RJHL, DL, WC, LR, ECR, RTPL, DCL, LFW, LN, BY.

*Statistical analysis:* XYP, IRL, SWF, YSG, AR, NW, ATR, BY.

*Obtained funding:* LR, DL, LFW, BY.

*Supervision:* LR, DCL, LFW, LN, BY.

A/Prof Young and Prof Ng had full access to all the data in the study and verified the data. A/Prof Young had final responsibility for the decision to submit for publication. All authors read and approved the final manuscript.

## Data sharing statement

An anonymised, de-identified version of the dataset can be made available upon request to allow all results to be reproduced.

## Declaration of interests

Young reports personal fees from Astra-Zeneca, Gilead, Moderna, Pfizer and Sanofi outside the submitted work. Chia and Wang disclose that a patent (Patent No.: US 11,054,429 B1) was issued for the surrogate virus neutralisation test platform (cPass kit) used in this study, and declare royalty payment from GenScript for inventing the technology. All other authors no potential conflicts of interest.

## Appendix A Supplementary data

Supplementary Methods, Figures and Tables

PRIBIVAC_Protocol_V11 dated 01 Aug 2023
