# Supplementary material for: First SARS-CoV-2 Omicron infection as an effective immune booster among mRNA vaccinated individuals: final results from the first phase of the PRIBIVAC randomised clinical trial
Source: eBioMedicine. 2024 Aug 12;107:105275. doi: 10.1016/j.ebiom.2024.105275 (PMC11367514; doi:10.1016/j.ebiom.2024.105275)
Supplement: Supplementary Methods, Figures and Tables [file mmc1.pdf]

Supplementary Materials

First SARS-CoV-2 Omicron infection as an effective immune booster among mRNA vaccinated individuals: Final results from the first phase of the PRIBIVAC randomized clinical trial

Xuan Ying Poh, I. Russel Lee1, Chee Wah Tan, Jean-Marc Chavatte, Siew Wai Fong, Yun Shan Goh, Angeline Rouers, Nathan Wong, Anthony Torres-Ruesta, Shirley Y.Y. Mah, Aileen Y.Y. Yeoh, Mihir Gandhi, Nabilah Rahman, Yi Qing Chin, J. Jonathan Lim, Terence J.K. Yoong, Suma Rao, Po Ying Chia, Sean W.X. Ong, Tau Hong Lee, Sapna P. Sadarangani, Ray J.H. Lin, Daniel R.X Lim, Wanni Chia, Laurent Renia, Ee Chee Ren, Raymond T.P. Lin, David C. Lye, Lin-Fa Wang, Lisa F.P. Ng, Barnaby E. Young

Table of Contents

Supplemental Method 1: Quantitative measurement of SARS-CoV-2-specific T cell responses

Supplemental Method 2: Quantitative measurement of SARS-CoV-2-specific memory B cell (MBC) responses

Supplemental Table 1. Surface and intracellular markers utilized for high-dimensional flow cytometry analysis of circulating T cell responses.

Supplemental Table 2. Longitudinal measurements of cytokine-expressing T cell subsets against wildtype SARS-CoV-2 peptides over a year of study period to examine vaccine- and infection-induced cellular immune responses.

Supplemental Fig. 1. SARS-CoV-2 anti-S-RBD titres in 61 participants who were COVID-19 naïve up to day 180.

Supplemental Fig. 2. SARS-CoV-2 full length spike antibody levels in 61 participants who were COVID-19 naïve up to day 180.

Supplemental Fig. 3. Qualitative detection of neutralising antibodies against wildtype SARS-CoV-2 among 61 participants who were COVID-19 naïve up to day 180.

Supplemental Fig. 4. Antibody response against wildtype SARS-CoV-2 at day 360 post-booster among 27 participants who remained uninfected.

Supplemental Fig 5. SARS-CoV-2 anti-S-RBD titres grouped by infection status/period (uninfected, early infection, late infection).

Supplemental Fig. 6. Longitudinal measurements of full-length SARS-CoV-2 Spike antibodies by SFB assay over a one-year study period to examine vaccine- and infection-induced humoral immune response.

Supplemental Fig. 7. Longitudinal measurements of antibody neutralization against wildtype SARS-CoV-2 over a one-year study period to examine vaccine- and infection-induced humoral immune response.

Supplemental Fig. 8. Longitudinal measurements of antibody neutralization against Omicron BA.1 over a one-year study period to examine vaccine-induced humoral immune response.

Supplemental Method 1: Quantitative measurement of SARS-CoV-2-specific T cell responses

Briefly, 2×10^6 frozen peripheral blood mononuclear cells (PBMCs) isolated from vaccinees were thawed and incubated in RPMI (Hyclone) with 5% human serum (Innovative Research) overnight at 37°C in 5% CO_2. PBMCs were stimulated the following day with either pooled PepTivator SARS-CoV-2 S and S1 peptides from the wildtype SARS-CoV-2 strain (0.6 nmol/mL each, Miltenyi Biotec), or a combination of phorbol-12-myristate-13-acetate (PMA; 100 ng/mL, Sigma-Aldrich) and ionomycin (1 µg/mL, Sigma-Aldrich) used as a positive control, or left unstimulated (baseline). PBMCs were incubated for 2 hours before a mixture of 1× Brefeldin A and 1× Monensin (Thermo Fisher Scientific) were added. PBMCs were further incubated for 4 hours at 37°C in 5% CO_2. Cells were washed with PBS and stained for surface markers (Supplemental Table 1) for 20 min at room temperature. PBMCs were washed, fixed and permeabilized for 20 min using the Foxp3/Transcription Factor Staining Buffer Set (Thermo Fisher Scientific). Cells were stained for intracellular cytokines (Supplemental Table 1) for 20 min before acquisition using a Cytek® Aurora cytometer (SpectroFlo® Version 2.2.0.3). Fluorescence-activated cell sorted (FACS) files were analyzed using FlowJo v10.8.2.

Supplemental Method 2: Quantitative measurement of SARS-CoV-2-specific memory B cell (MBC) responses

Briefly, MultiScreen HTS IP Filter Plate, 0·45 µm (Merck Millipore) was coated with purified anti-human-IgG (15 µg/mL in PBS, MT91/145, Mabtech) or SARS-CoV-2 wildtype RBD protein (10 µg/mL, NUS Life Sciences Institute) in 1× PBS at 4°C overnight. Plates were washed and blocked for 30 min with RPMI + 10% FBS at room temperature. 1×10^6 PBMCs isolated from vaccinees were resuspended in 1 mL RPMI + 10% FBS +1 µg/mL R848 + 10 ng/mL recombinant human IL-2, and incubated at 37°C, 5% CO_2 for 5 days for the MBC to differentiate into antibody-secreting cells (ASC). After the 5 days, $1–4 \times 10^5$ live ASCs were used to determine RBD-specific MBC numbers by ELISpot. Total IgG-secreting cells were determined by plating $1.5–3 \times 10^3$ live cells. Cells were incubated for 18–22 hours at 37°C with 5% CO_2 in the ELISpot plate before detection. Anti-IgG biotinylated/streptavidin-ALP (Mabtech) was used to detect RBD-specific and total IgG-secreting cells. Plates were then read on an IRIS ELISpot reader (Mabtech).

Supplemental Table 1. Surface and intracellular markers utilized for high-dimensional flow cytometry analysis of circulating T cell responses.

Surface marker	Fluorophore	Clone	Catalogue #	Company
CD8	BV605	SK1	564116	BD Biosciences
CD19	BV605	HIB19	740394	BD Biosciences
VD2	BV711	B6	331412	Biolegend
CD107a	BV785	H4A3	563869	BD Biosciences
CD3	SB550	SK7	344852	Biolegend
CD4	SN685	SK3	344658	Biolegend
VD1	APC Vio770	REA173	130-120-578	Miltenyi Biotec
Live/Dead	Zombie NIR	-	423105	Biolegend
CXCR5	PE Vio615	J252D4	356928	Biolegend
CD154	PE-Cy5	TRAP-1	555701	BD Biosciences
CD45	BUV805	H130	612891	BD Biosciences
**Intracellular marker**	**Fluorophore**	**Clone**	**Catalogue #**	**Company**
Granzyme B	BV510	GB11	563388	BD Biosciences
IFNγ	BV570	4S.B3	502534	Biolegend
TNFα	BV750	MAB11	566359	BD Biosciences
IL-2	BV650	MQ1-17H12	564166	BD Biosciences
IL-4	PE-Cy7	8D4-8	560672	BD Biosciences
IL-10	PE	JES-9D7	501404	Biolegend
IL-17a	AF647	N49-653	560490	BD Biosciences

Supplemental Table 2. Longitudinal measurements of cytokine-expressing T cell subsets against wildtype SARS-CoV-2 peptides over a year of study period to examine vaccine- and infection-induced cellular immune responses. Data was analysed using Kruskal-Wallis test with Dunn's multiple comparisons test.

***p* value**	**Uninfected vs Early infection**	**Uninfected vs Late infection**	**Early infection vs Late infection**
**IFNγ+ CD4+ T cells**			
**Day 0**	>0.99	>0.99	>0.99
**Day 7**	>0.99	>0.99	>0.99
**Day 28**	0.73	0.43	>0.99
**Day 180**	0.63	>0.99	0.33
**Day 360**	>0.99	0.31	>0.99
**IL-2+ CD4+ T cells**			
**Day 0**	>0.99	>0.99	>0.99
**Day 7**	>0.99	>0.99	>0.99
**Day 28**	>0.99	>0.99	>0.99
**Day 180**	>0.99	>0.99	>0.99
**Day 360**	0.98	>0.99	0.82
**TNFα+ CD4+ T cells**			
**Day 0**	>0.99	0.42	0.37
**Day 7**	>0.99	>0.99	>0.99
**Day 28**	0.73	>0.99	>0.99
**Day 180**	>0.99	0.72	>0.99
**Day 360**	>0.99	>0.99	>0.99
**IL-4+ CD4+ T cells**			
**Day 0**	>0.99	>0.99	>0.99
**Day 7**	>0.99	>0.99	>0.99
**Day 28**	0.63	>0.99	0.82
**Day 180**	>0.99	>0.99	>0.99
**Day 360**	>0.99	>0.99	>0.99
**IL-6+ CD4+ T cells**			
**Day 0**	>0.99	>0.99	>0.99
**Day 7**	0.84	>0.99	0.40
**Day 28**	>0.99	0.71	0.22
**Day 180**	>0.99	>0.99	>0.99
**Day 360**	>0.99	>0.99	>0.99
**IL-17α+ CD4+ T cells**			
**Day 0**	0.47	>0.99	>0.99
**Day 7**	>0.99	>0.99	>0.99
**Day 28**	>0.99	>0.99	0.99
**Day 180**	>0.99	0.83	>0.99
**Day 360**	>0.99	>0.99	>0.99
**Granzyme B+ CD8+ T cells**			
**Day 0**	>0.99	>0.99	>0.99
**Day 7**	>0.99	>0.99	>0.99
**Day 28**	0.11	>0.99	0.29
**Day 180**	>0.99	0.66	0.26
**Day 360**	>0.99	>0.99	>0.99
**IFNγ+ CD8+ T cells**			
**Day 0**	>0.99	>0.99	>0.99
**Day 7**	>0.99	>0.99	>0.99
**Day 28**	0.32	0.63	>0.99
**Day 180**	0.89	0.72	>0.99
**Day 360**	>0.99	0.80	>0.99

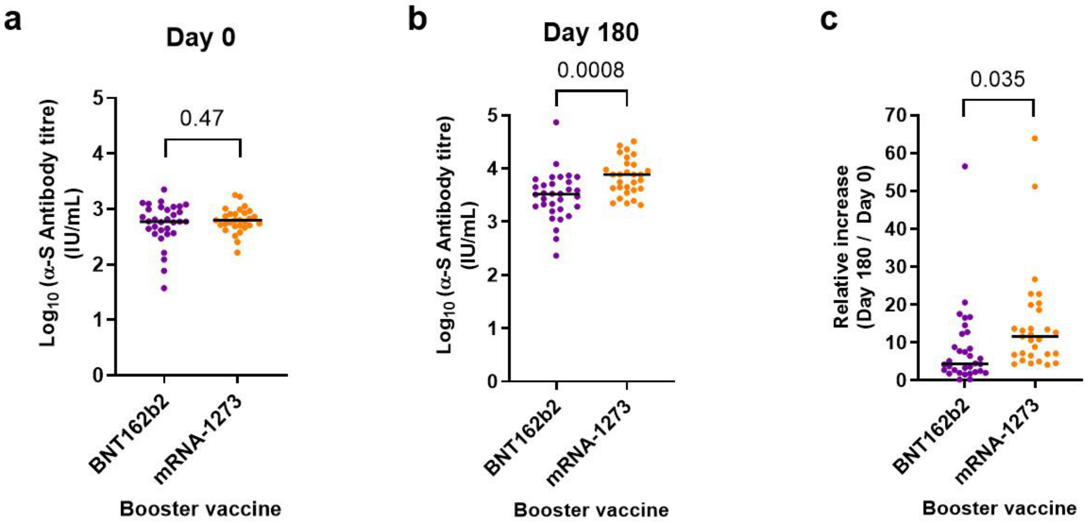

Supplemental Fig. 1. SARS-CoV-2 anti-S-RBD titres in 61 participants who were COVID-19 naïve up to day 180. Mean SARS-CoV-2 anti-S-RBD titres between homologous (BNT162b2) and heterologous (mRNA-1273) groups are presented at (a) pre-booster day 0 and (b) day 180 post-booster. (c) The relative increase (at day 180) from baseline between vaccine groups. Data was analysed using Student *t* test. Each dot represents data from one participant.

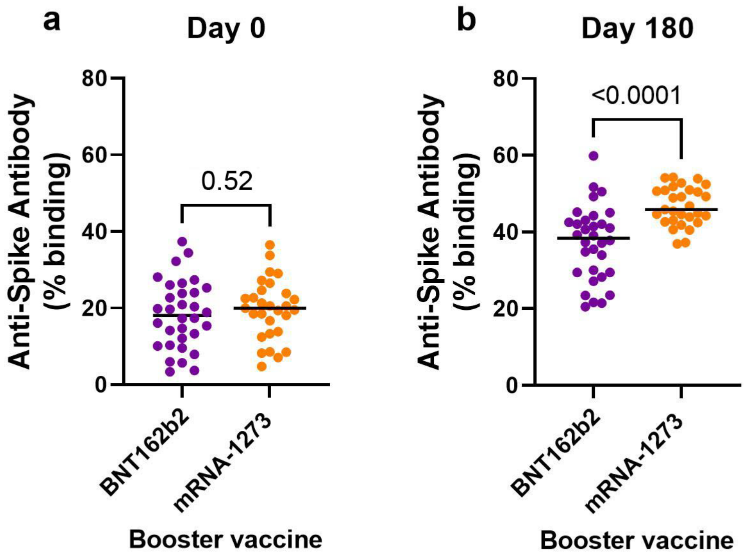

Supplemental Fig. 2. SARS-CoV-2 full length spike antibody levels in 61 participants who were COVID-19 naïve up to day 180. Levels of SARS-CoV-2 anti-S immunoglobulins between homologous (BNT162b2) and heterologous (mRNA-1273) groups are presented at (a) pre-booster day 0 and (b) day 180 post-booster. Data was analysed using Mann-Whitney U test. Each dot represents data from one participant.

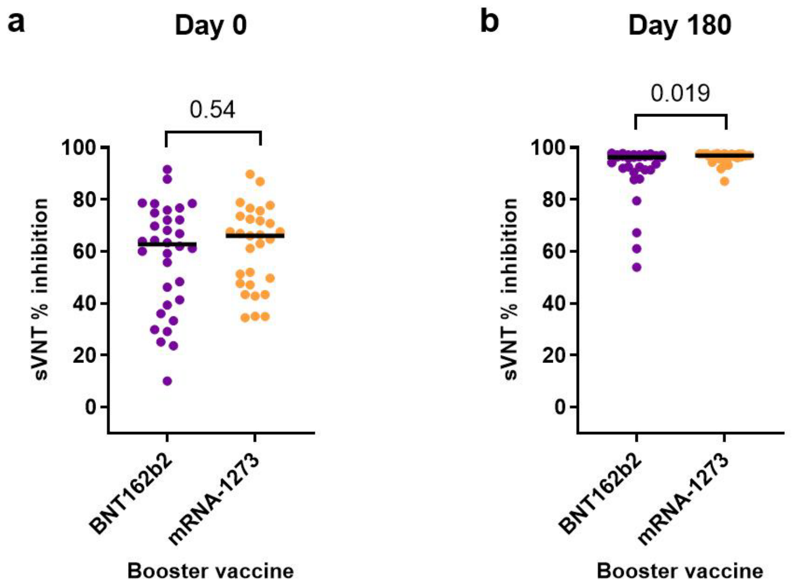

Supplemental Fig. 3. Qualitative detection of neutralising antibodies against wildtype SARS-CoV-2 among 61 participants who were COVID-19 naïve up to day 180. Median SARS-CoV-2 neutralising antibody levels between homologous (BNT162b2) and heterologous (mRNA-1273) groups are presented at (a) pre-booster day 0 and (b) day 180 post-booster. The nomimal "seronegative" threshold is 30%, where inhibition of <30% indicates an absence of SARS-CoV-2 neutralising antibodies or level of SARS-CoV-2 neutralising antibodies below the sVNT limit of detection. Data was analysed using Mann-Whitney U test. Each dot represents data from one participant.

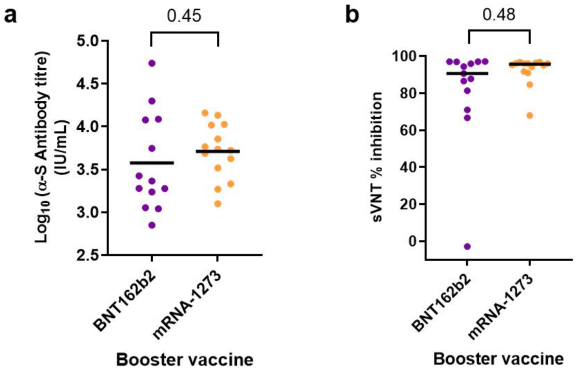

Supplemental Fig. 4. Antibody response against wildtype SARS-CoV-2 at day 360 post-booster among 27 participants who remained uninfected. (a) Anti-S-RBD titres and (b) neutralising antibody titres are presented. Data was analysed using Student *t* test and Mann-Whitney U test for anti-S-RBD titres and neutralising antibody levels respectively. Each dot represents data from one participant.

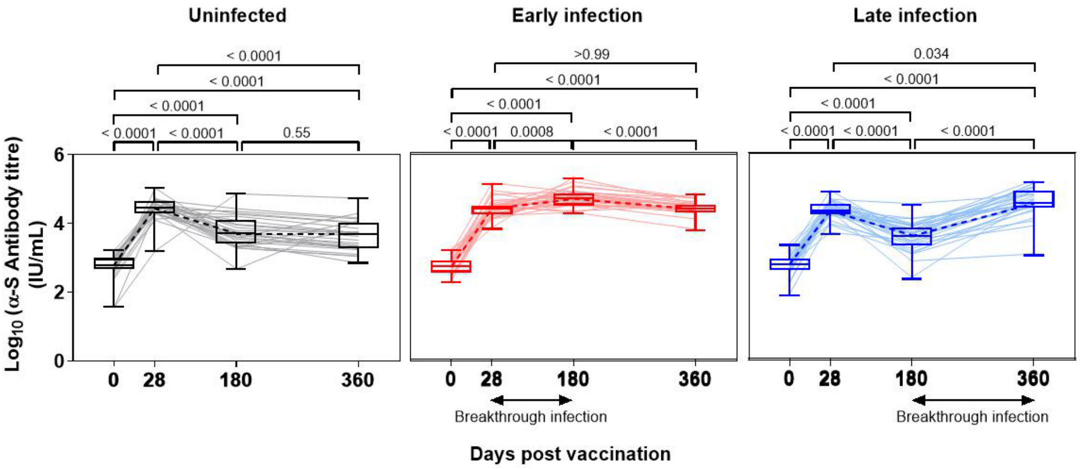

Supplemental Fig 5. SARS-CoV-2 anti-S-RBD titres grouped by infection status/period (uninfected, early infection, late infection). Paired samples across the timepoints were analysed using RM one-way ANOVA with Tukey's multiple comparison test.

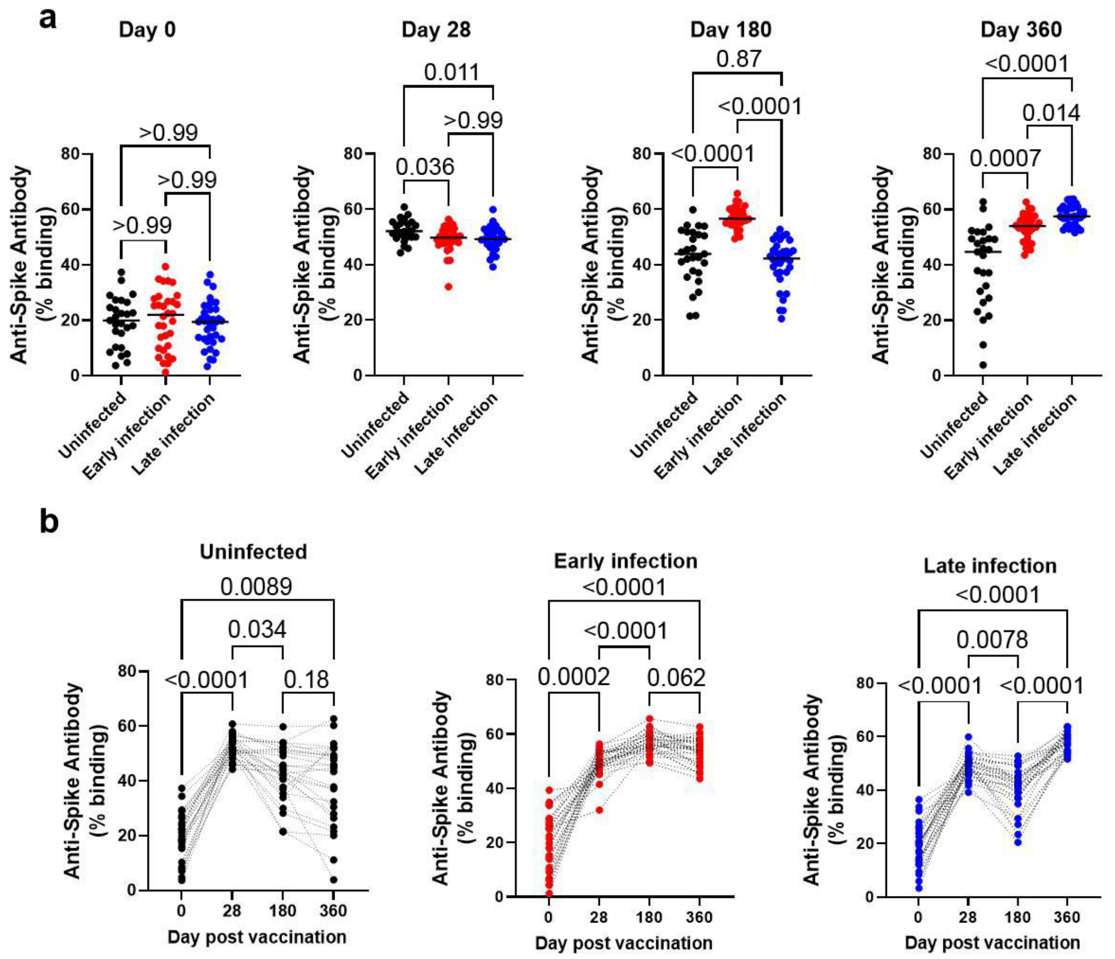

Supplemental Fig. 6. Longitudinal measurements of full-length SARS-CoV-2 Spike antibodies by SFB assay over a one-year study period to examine vaccine- and infection-induced humoral immune response. Participants (*n* = 93) had the BNT162b2 primary vaccination series prior to enrolment, and received either BNT162b2 or mRNA-1273 booster at study day 1. Within the cohort, 34.4% (32/93) were COVID-19 infected from days 29–180 and 36.6% (34/93) from days 181–360. Each dot represents data from one participant. a) Data grouped by study visits (day 0 [pre-booster], post-booster days 28, 180 and 360). Data was analysed using Kruskal-Wallis test with Dunn's multiple comparison test. (b) Data grouped by infection status/period (uninfected, COVID-19 between days 29 and 180, COVID-19 between days 181 and 360). Paired samples across the timepoints were analysed using Friedman test with Dunn's multiple comparison test.

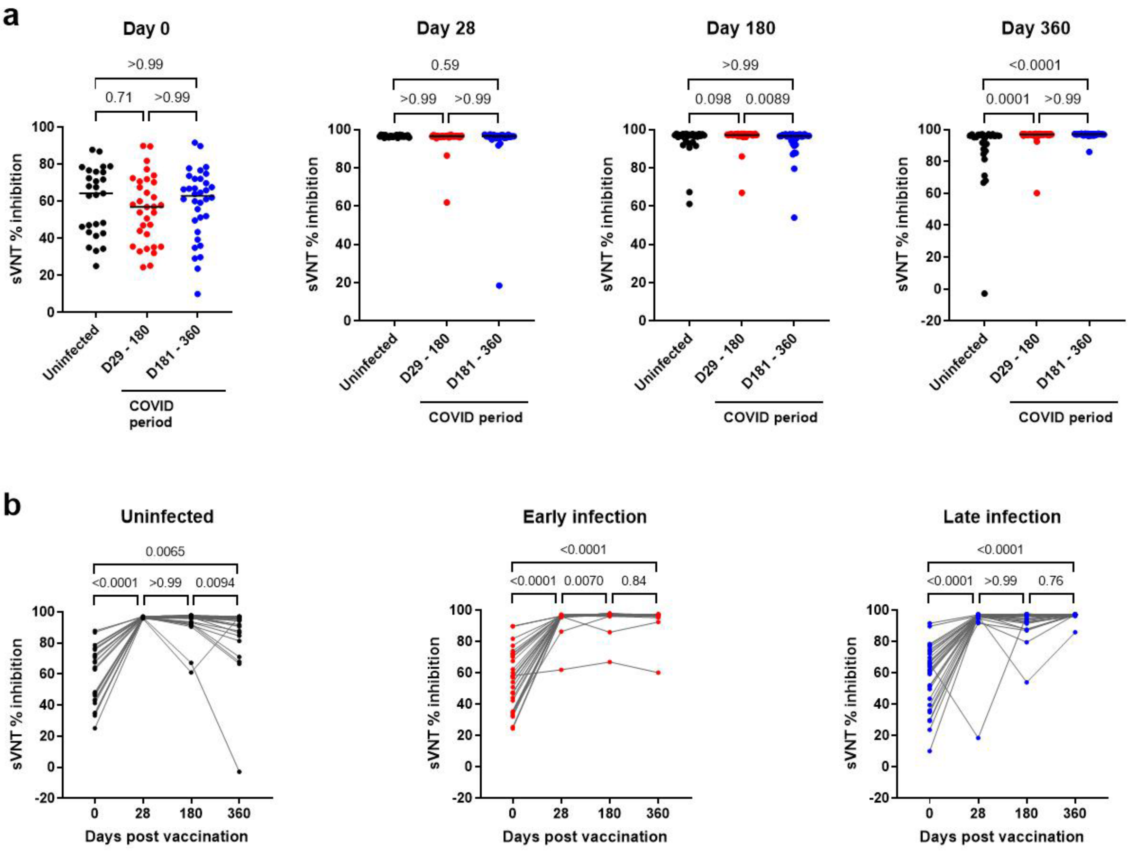

Supplemental Fig. 7. Longitudinal measurements of antibody neutralization against wildtype SARS-CoV-2 over a one-year study period to examine vaccine- and infection-induced humoral immune response. Participants (*n* = 93) had the BNT162b2 primary vaccination series prior to enrolment, and received either BNT162b2 or mRNA-1273 booster at study day 1. Within the cohort, 34.4% (32/93) were COVID-19 infected from days 29–180 and 36.6% (34/93) from days 181–360. Each dot represents data from one participant. (a) Data grouped by study visits (day 0 [pre-booster], post-booster days 28, 180 and 360). Bar represents median. Data was analysed using Kruskal-Wallis test with Dunn's multiple comparison test. (b) Data grouped by infection status/period (uninfected, COVID-19 between days 29 and 180, COVID-19 between days 181 and 360). Paired samples across the time points were analysed using Friedman test with Dunn's multiple comparison test.

a

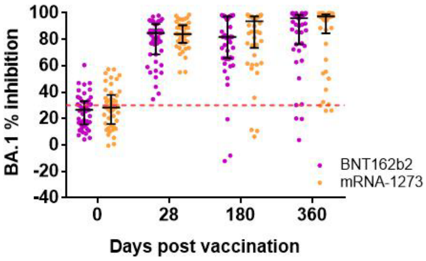

b

		BNT162b2	mRNA-1273	*p* value
**Day**	**N**	47	46	
**0**	**Median [IQR] (%)**	26.5 [15.5-33.1]	28.3 [15.8-37.3]	0.30
**Day**	**N**	47	44	
**28**	**Median [IQR] (%)**	84.6 [68.6-91.5]	84 [77.3-90.8]	0.32
**Day**	**N**	47	46	
**180**	**Median [IQR] (%)**	81.9 [65.8-97.4]	93.7 [73.5-97.4]	0.39
**Day**	**N**	47	46	
**360**	**Median [IQR] (%)**	95.9 [76.3-98.7]	97.4 [84.5-99.0]	0.35

Supplemental Fig. 8. Longitudinal measurements of antibody neutralization against Omicron BA.1 over a one-year study period to examine vaccine-induced humoral immune response. (a) BA.1 sVNT inhibition level between homologous (BNT162b2) and heterologous (mRNA-1273) vaccine arms. Data was analysed using Mann–Whitney U test. (b) Summary data.
