# Supplementary material for: First SARS-CoV-2 Omicron infection as an effective immune booster among mRNA vaccinated individuals: final results from the first phase of the PRIBIVAC randomised clinical trial
Source: eBioMedicine. 2024 Aug 12;107:105275. doi: 10.1016/j.ebiom.2024.105275 (PMC11367514; doi:10.1016/j.ebiom.2024.105275)
Supplement: PRIBIVAC_Protocol_V11 dated 01 Aug 2023 [file mmc2.pdf]

STUDY PROTOCOL

PROTOCOL TITLE:
Heterologous prime-boost-boost vaccine combinations for long-term humoral and cellular immunity against COVID-19 [PRIBIVAC]

PROTOCOL NUMBER:
2021/00821

PROTOCOL VERSION: 11
PROTOCOL DATE: 01 August 2023

PRINCIPAL INVESTIGATOR:

Dr Barnaby Young
Senior Consultant, National Centre for Infectious Diseases (NCID), Singapore
Email: barnaby_young@ncid.sg

CO-INVESTIGATORS:

Prof David Lye
Senior Consultant and Director, Infectious Disease Research and Training Office, NCID
Email: David_Lye@ncid.sg

Dr Sapna Sadarangani
Consultant, NCID, Singapore
Email: sapna_sadarangani@ncid.sg

Dr Lin Junhao Ray
Consultant, NCID, Singapore
Email: ray_jh_lin@ttsh.com.sg

Dr Chia Po Ying
Consultant, NCID, Singapore
Email: po_ying_chia@ncid.sg

Dr Suma Rao
Consultant, NCID, Singapore
Email: suma_rao@ncid.sg

Dr Sean Ong Wei Xiang
Senior Resident

Email: sean.ongwx@mohh.com.sg

COLLABORATORS:

Prof Laurent Renia
A*STAR Infectious Diseases Labs
Email: Renia_Laurent@IDLabs.a-star.edu.sg

Prof Lisa Ng
A*STAR Infectious Diseases Labs
Email: Lisa_Ng@IDLabs.a-star.edu.sg

A/Prof Ren Ee Chee
Singapore Immunology Network, A*STAR
Email: Ren_Ee_Chee@immunol.a-star.edu.sg

Prof Linfa Wang
Duke-NUS Medical School, Duke-NUS
Email: linfa.wang@duke-nus.edu.sg

A/Prof Raymond Lin
Senior Consultant and Director, National Public Health Laboratory, NCID
Email: Raymond_Lin@ncid.sg

Prof Jerry Chan
KK Women's and Children's Hospital
Email: jerry.chan.k.y@singhealth.com.sg

STUDY SITES:

National Centre for Infectious Diseases, 16 Jalan Tan Tock Seng, Singapore 308442
A*star ID labs, 8A Biomedical Grove, Immunos Building, Singapore 138648

TABLE OF CONTENTS

1. BACKGROUND AND RATIONALE 5

1.1. GENERAL INTRODUCTION 5
1.2. RATIONALE AND JUSTIFICATION FOR THE STUDY 5
A. RATIONALE FOR THE STUDY PURPOSE 5
B. RATIONALE FOR DOSES SELECTED 8
C. RATIONALE FOR STUDY POPULATION 8
D. RATIONALE FOR STUDY DESIGN 9

2. HYPOTHESIS AND OBJECTIVES 10

2.1. HYPOTHESIS 10
2.4. POTENTIAL RISKS AND BENEFITS: 12
A. END POINTS - EFFICACY 12
B. END POINTS - SAFETY 12

3. STUDY POPULATION 5

3.1. LIST THE NUMBER OF SUBJECTS TO BE ENROLLED. 12
3.2. CRITERIA FOR RECRUITMENT 13
3.3. INCLUSION CRITERIA 13
3.4. EXCLUSION CRITERIA 13
3.5. WITHDRAWAL CRITERIA 14
3.6. SUBJECT REPLACEMENT 14

4. TRIAL SCHEDULE 14

5. STUDY DESIGN 6

5. STUDY DESIGN 16
5.1. SUMMARY OF STUDY DESIGN 16

6. METHODS AND ASSESSMENTS 17

6.1. RANDOMISATION AND BLINDING 17
6.2. CONTRACEPTION AND PREGNANCY TESTING 18
6.3. STUDY VISITS AND PROCEDURES 18

7. TRIAL MATERIALS 20

7.1. TRIAL PRODUCT (S) 20
7.2. STORAGE AND DRUG ACCOUNTABILITY 21

8. TREATMENT 21

8.1. RATIONALE FOR SELECTION OF DOSE 21
8.2. STUDY DRUG FORMULATIONS 21
8.3. STUDY DRUG ADMINISTRATION 22
8.4. SPECIFIC RESTRICTIONS / REQUIREMENTS 22
8.5. BLINDING 22
8.6. CONCOMITANT THERAPY 22

9. SAFETY MEASUREMENTS 23

9.1. DEFINITIONS 23
9.2. COLLECTING, RECORDING AND REPORTING OF "UNANTICIPATED PROBLEMS INVOLVING RISK TO SUBJECTS OR OTHERS" – UPIRTSO EVENTS TO THE NHG DOMAIN SPECIFIC REVIEW BOARDS (DSRB) 23
9.3. COLLECTING, RECORDING AND REPORTING OF SERIOUS ADVERSE EVENTS (SAES) TO THE HEALTH SCIENCE AUTHORITY (HSA) 24

9.4. Safety Monitoring Plan 24
9.5. Complaint Handling – 25

10. DATA ANALYSIS 25

10.1. Data Quality Assurance 25
10.2. Data Entry and Storage 25

11. SAMPLE SIZE AND STATISTICAL METHODS 25

11.1. Determination of Sample Size 25
11.2. Statistical and Analytical Plans 26

12. ETHICAL CONSIDERATIONS 27

12.1. Informed Consent 27
12.2. IRB review 28
12.3. Confidentiality of Data and Patient Records 28

13. PUBLICATIONS 28

14. RETENTION OF TRIAL DOCUMENTS 28

STUDY PROTOCOL

1. BACKGROUND AND RATIONALE

1.1. General Introduction

In December 2019, a novel coronavirus (named severe acute respiratory syndrome coronavirus 2; SARS-CoV-2) emerged from Wuhan, China, as the cause of a transmissible respiratory tract infection in humans.[1] On 11 March 2020, the World Health Organization (WHO) declared COVID-19 a global pandemic.[2] By August 2021 more than 200 million confirmed cases had been reported worldwide and >4.4 million deaths, while in Singapore there had been >66,000 cases and >50 deaths.

The COVID-19 global health crisis has spurred vaccine development and phase 1/2/3 clinical trials in record time.[3] As of 23 Aug 2021, the WHO reported 139 vaccine candidates being investigated in 415 phase 1-3 trials and 22 that have been approved for clinical use in at least one country.[4] In Singapore two mRNA vaccines developed by Pfizer/BioNTech (BNT162b2) and Moderna (mRNA-1273), were granted interim authorisation by HSA under the pandemic special access route (PSAR) by the beginning of 2021. The pivotal phase 3 clinical trials for these vaccines reported efficacy of >95% against symptomatic infection and severe disease.[5,6] Some other COVID-19 vaccines are authorised by HSA for use in Singapore as part of the Special Access Route (SAR) through their addition to the WHO Emergency Use List (EUL). Many of these vaccines have been widely used in other countries, including an inactivated whole virus vaccine developed by Sinovac;[7] an adenovirus-based formulation developed by Oxford-AstraZenaca;[8] and a recombinant, replication-incompetent adenovirus serotype 26 (Ad26) vector vaccine developed by Johnson & Johnson / Janssen.[9]

COVID-19 vaccination programs worldwide have focused on raising population immunity through the primary COVID-19 vaccine series. However, eradication of the SARS-CoV-2 virus from humans is highly unlikely and vaccine breakthrough infections occur with increasing frequency as uptake increases. In addition, waning antibody levels are biologically inevitable following vaccination, and the emergence of variants of concern (VOC) capable of evading protective immunity (from a wildtype SARS-CoV-2 virus vaccine) has raised the need for a long term COVID-19 immunisation strategy. Several fundamental questions need to be addressed in order to design this: Who needs a booster vaccination? How long after the primary series should it be administered? And, which vaccine should be used?

1.2. Rationale and justification for the Study

a. Rationale for the Study Purpose

In Singapore, novel mRNA vaccines produced by Pfizer-BioNTech (BNT162b2) and Moderna (mRNA-1273) were the first COVID-19 vaccines to receive interim authorisation under the Pandemic Special Access Route (PSAR). Vaccination started first for frontline and healthcare workers with BNT162b2 at the end of December 2020. Over the following months this vaccine program was extended first to older adults and then the general population, while mRNA-1273 was also introduced alongside BNT162b2. The Sinovac-CoronaVac inactivated COVID-19 vaccine was added to the Singapore National Vaccination Programme in October 2021. In January 2022 vaccination was extended to children aged 5-11 years, and to date 96% of the eligible population in Singapore has completed their primary vaccine series. In Singapore ~97%

of vaccinated individuals have received an mRNA vaccine and 3% an inactivated vaccine (2% Sinovac-Coronavac, 1% Sinopharm BBIBP).

The pivotal phase 3 clinical trials of BNT162b2 and mRNA-1273 reported a vaccine efficacy of >95% against symptomatic and severe disease. However, waning antibody levels post-vaccination are inevitable and may also result in waning vaccine efficacy. Six-month follow up data for the mRNA-1273 vaccine estimated the half-life for binding antibodies for all participants was 52 days (95% CI 46 to 58), with significantly lower titres with increasing age.[10] Similarly long term data from the BNT162b2 phase 3 clinical trial described a decline in VE from 96.2% (95% CI 93.3 to 98.1) 7 days to < 2 months post-dose 2, to a VE of 90.1% (95% CI 86.6 to 92.9) from 2 months to <4 months, and 83.7% (95% CI 74.7 to 89.9) from 4 months to the data cut-off.[11] The Ministry of Health in Israel estimated that vaccine protection against infection declined from 90% at the beginning of 2021 to around 40% by late June.[12] Similar real world evidence has been reported from the UK and US.[13]

Some of the explanation for waning vaccine effectiveness is the emergence of SARS-CoV-2 variants of concern (VOC) which partially evade host immunity from prior infection/vaccination. This includes B.1.1.7 (Alpha), B.1.351 (Beta), P.1 (Gamma) and B.1.617.2 (Delta).[14] Studies in Singapore have documented that the mRNA vaccines retain high effectiveness against severe infection with Delta, however, higher antibody levels as measured against the 'wildtype' spike protein are likely to be necessary to protect against infection by Delta.[15,16] Given that eradication of SARS-CoV-2 is not possible with currently available vaccines a long-term vaccination strategy is required.

Clinical trials of vaccine candidates which target VOCs are ongoing, though even if successful these vaccines are not expected to be available till late in 2022. Pending the availability of these VOC vaccines, the need for booster vaccinations, particularly in vulnerable populations needs to be assessed. A third dose of the vaccines developed by Pfizer–BioNTech, Moderna, Oxford–AstraZeneca and Sinovac prompted significant increases in neutralizing antibodies titres when administered several months after the second dose.[17] However, there is limited data as to whether a homologous booster regimen can enhance protection against emerging VOC. Preliminary data from Israel (who have administered a third dose to 1.5 million individuals, ~15% of the population) reported a third dose boosted vaccine effectiveness in adults aged >60 years.[18] Preclinical data have also suggested heterologous prime-boost vaccination may enhance the immune response in COVID and HIV infections.[19-23] To date, there is only one phase 1/2 trial and one phase 3 trial published on heterologous prime-boost vaccination for COVID-19.[24,25] Both of these trials utilised an adenovirus-based COVID-19 vaccine (Gam-COVID-Vac, Sputnik V), which was shown to produce relatively low levels of neutralising antibodies in the phase 1/2 trial but has high efficacy of ~92% in the phase 3 trial.[24,25] Clinical trials are ongoing in the US and the UK comparing the effects of homologous and heterologous booster regimens. Due to the large number of potential vaccine combinations, the variation in vaccine types used by programmes in different countries, and potentially confounding effects of background infection rates, data relevant to Singapore's programme is not available.

The emergence of the highly transmissible Omicron VOC in November 2021 heralded a shift in booster vaccination policy in Singapore and overseas. First, recommendations for a third dose of an mRNA vaccine were strengthened and in Singapore receipt of a booster dose within 9 months of completing the primary vaccine series was mandated for maintenance of vaccinated status. Following these efforts booster vaccination uptake increased markedly, and currently

79% of the total population in Singapore has received their first booster vaccination dose. Secondly, recommendations changed to advise that individuals complete the standard vaccination regimen even if they have had prior COVID-19. Thirdly, attention shifted to providing second boosters among adults who at highest risk of severe COVID.

Although the original monovalent COVID-19 vaccines have proven effective at preventing death and severe disease, breakthrough infections and reinfections have become more common in the face of an evolving virus. The ever-mutating Omicron virus has resulted in several sublineages such as BA.1, BA.2, BA.4 and BA.5 that have caused surges in COVID-19 cases worldwide. Lab studies consistently suggest that antibodies triggered by vaccines (targeting the ancestral SARS-CoV-2 strain) are less effective at blocking BA.4 and BA.5 than earlier Omicron strains BA.1 and BA.2.[26-30] This could leave previously infected and/or vaccinated and boosted individuals vulnerable to multiple Omicron infections. Thus, pharmaceutical companies have moved towards developing variant-based vaccines to ensure individuals maintain a high level of protection. On 31 August 2022, the US FDA authorized the bivalent formulations of the Moderna and Pfizer-BioNTech vaccines for use as a single booster dose at least two months following primary or booster vaccination.[31] Both bivalent vaccines from Moderna and Pfizer-BioNTech target the ancestral SARS-CoV-2 strain from 2020 and the Omicron variant BA.4/5 lineages.[31] Preclinical studies have shown that boosting with bivalent Omicron-based mRNA-1273.214 (targeting BA.1 subvariant) or mRNA-1273.222 (targeting BA.4/5 subvariant) increases breadth of neutralization and confers protection in mice against the currently circulating BA.5 Omicron variant.[32] An open-label, ongoing phase 2/3 study comparing the safety and immunogenicity of the bivalent Omicron-containing vaccine mRNA-1273-214 with the monovalent vaccine mRNA-1273 as a second booster in 819 adults demonstrated that the bivalent mRNA-1273.214 booster vaccine was safe and elicited superior neutralizing antibody responses against Omicron than mRNA-1273.[33] Clinical trials for mRNA-1273.222 and Comirnaty Bivalent (Original/Omicron BA.4/5) vaccines are still under-way.

Since the first detection of the XBB Omicron subvariant in August 2022 in India, it has been detected in more than 17 countries to-date including Singapore, Australia, Bangladesh, Denmark, Japan and the US. The current wave of COVID-19 cases in Singapore, driven by the XBB strain, is expected to peak in mid-November 2022 with about 15,000 daily cases on average. The proportion of reinfections among total COVID-19 cases in Singapore is also on the rise, currently making up about 17% of total new cases. To combat the rising COVID-19 infections and reinfections, the bivalent Moderna/Spikevax vaccine was added to the National Vaccination Programme from 14 October 2022 for persons who have yet to achieve minimum protection (i.e. received at least 3 doses of mRNA/Nuvaxovid or 4 doses of Sinovac-Coronavac), or those aged 50 years and above who have received their last vaccine dose more than five months ago. On 25 October 2022, Singapore's HSA granted interim authorisation of a booster dose of the Pfizer-BioNTech bivalent vaccine for individuals aged 12 years and older.

From 14 Nov 2022, a second booster dose from five months to one year after the last vaccine dose is recommended by MOH for all individuals aged 18 years or older.

The updated bivalent vaccine is preferred for this. A person is considered up-to-date with their COVID-19 vaccination if they have received at least the minimum protection and their last vaccine dose was received within the past one year. The bivalent Moderna/Spikevax vaccine is currently available, and the bivalent Pfizer-BioNTech vaccine is expected to be available by the end of this year.

PRIBIVAC will assess a heterologous prime-boost-boost strategy in comparison with a homologous regimen in order to compare short and long-term immunogenicity of different COVID-19 vaccine combinations against the ancestral SARS-CoV-2 as well as different VOCs. Initial phases of the study have studied homologous versus heterologous vaccines at the first booster, later phases will study these as the second booster.

Third-dose boosters increase humoral and cellular immunity,[34] but the rapid waning of protection against symptomatic infection with VOCs such as Omicron[35] has prompted countries to call for a fourth-dose vaccination program. However, the clinical need, effectiveness and timing of a fourth-dose vaccine booster remain to be assessed. As of March 2022, Israel[36] and Germany[37] began offering fourth-dose booster vaccines to their populations. Observational data from Israeli health care workers have shown a boosting effect on immunogenicity after a fourth-dose of mRNA vaccine, but the low vaccine efficacy against infections suggested that a fourth-dose vaccination in young health care workers may have only marginal benefits.[36] Restoration of anti-Spike antibody levels after a fourth-dose mRNA vaccine was similarly observed in a German observational study, although the neutralising capacity against Omicron subvariants BA.1 and BA.2 remained low.[37] This suggests that at-risk individuals such as immunocompromised patients or the elderly who have prominent immune waning[38] and high risk for severe disease are more likely to benefit from additional vaccine boosters, particularly during times of high SARS-CoV-2 incidence. In April 2022, the UK rolled out fourth doses for clinically vulnerable populations (COV-BOOST trial) and showed that a fourth dose mRNA vaccine boosted both cellular and humoral immunity to similar, or higher, levels seen at 28 days after a third dose.[39]

Over 2020-22, our research team in Singapore and others have investigated the nature and duration of COVID-19 immune responses, and tried to uncover immune correlates of protection.[40] This led to the development of validated immunological assays, including antibody and T-cell based assays, to identify individuals who have cleared the SARS-CoV-2 infection and developed protective immunity. These assays are also being used for development and assessment of various vaccine candidates. They will help to precisely define the immunogenicity of the different prime-boost-boost vaccine combinations in various populations. Additionally, the assays will help to determine whether the 'doctrine of original antigenic sin' is observed in prime-boost-boost vaccine combinations.

b. Rationale for Doses Selected

Different types of COVID-19 vaccines have been purchased by Singapore and are undergoing evaluation for licensure. Authorisation for the use of these vaccines in this study will be sought from Health Sciences Authority (HSA) and Ministry of Health (MOH). The vaccine doses for each brand will be according to manufacturer's instructions.

c. Rationale for Study Population

In the early phases of this study (Phase A to C) volunteers who previously received a homologous primary vaccine series with BNT162b2 (Comirnaty/Pfizer-BioNTech) or mRNA-1273 (Moderna) were enrolled.[41] With robust data now available about the benefits of a first booster dose of the vaccine, phase D of the study will shift to investigating the second booster dose and will simplify eligibility criteria to reflect heterogeneity in vaccinations received among

the Singapore population to date, and the high prevalence of COVID-19 infection during the Omicron wave.

Circulating antibody levels wane following vaccination and COVID-19 infection. In our previous study of COVID-19 recovered patients, ~60% of the cohort (n = 164) retained >30% inhibition level of neutralising antibodies against SARS-CoV-2 at 6 months post infection.[16] Data from individuals vaccinated with mRNA-1273 showed gradually declining neutralising antibody titres by 6 months post inoculation.[10] This waning of antibody levels also occurs following the first booster.

Potential participants for PRIBIVAC will be invited and volunteers are followed at several time points post-immunisation for up to 12 months for assessment of cellular immune parameters.

d. Rationale for Study Design

PRIBIVAC is an adaptive, randomised, subject-blinded, controlled trial to assess the immunogenicity and safety of heterologous boost COVID-19 vaccine regimens compared with a homologous boost regimen. Potential participants would have already received a primary vaccine series with BNT162b2 or mRNA-1273 prior to randomisation.

In Phases A-C of this study, the booster vaccine for the control group was the homologous mRNA vaccine (e.g. BNT162b2 + BNT162b2 + BNT162b2 or mRNA-1273 + mRNA-1273 + mRNA-1273), while the heterologous vaccine candidates were an alternative COVID-19 vaccine. For individuals randomised to intervention group 1 the mRNA booster vaccine administered was heterologous to the primary series (e.g. BNT162b2 + BNT162b2 + mRNA-1273 or mRNA-1273 + mRNA-1273 + BNT162b2), while for other intervention groups this included vaccines of alternate designs:

Control group: Homologous mRNA booster vaccine
Intervention group 1: Heterologous mRNA booster vaccine (Phase A)
Intervention group 2: COVAXIN® (Phase B)
Intervention group 3: Nuvaxovid (Phase C)

Vaccine candidates in phases A-C entered the study at different time points and participants were randomised at equal probability to the available intervention arms at the time of randomisation. This will reduce the risk of bias (e.g. participant preference for a certain arm) compared with a non-randomised design. While an ideal scenario is to have all the selected vaccines approved prior to the start of recruitment, due to the unknown time of availability of vaccine candidates A, B and C, and the urgency of the current situation, recruitment to the control and intervention group 1 (Phase A) was initiated from 12 Oct 2021. Intervention group 2 (Phase B) was added in January 2022 and discontinued 15 March 2022 per the recommendation from the Data Safety Monitoring Board, who reviewed the trial's preliminary results from the first 10 COVAXIN recipients up to Day 28 post-booster. Recruitment to Intervention group 3 (Phase C) was terminated prior to initiation due to delays in the availability of Nuvaxovid which despite receiving interim authorisation by HSA in February 2022 has only become available for administration in late May 2022.

As of 18-May-2022 uptake of the first booster has increased to 75% of the population – with a sharp increase in uptake from January 2022 following governmental policy changes to the definitions of vaccinated status. A significant proportion of those who have not taken their

booster are expected to have either had COVID-19 during the Omicron wave, which severely restricts the ability of the study to continue as initially designed. Related to this, focus is shifting to longer term strategies with a second COVID-19 vaccine booster program beginning for selected individuals in Singapore in March 2022. Phase D of the study will examine this immune response following a second COVID-19 vaccine booster. Study participants will be randomised to receive one of three vaccines in equal proportions: Comirnaty or Cormirnaty Bivalent (Original/Omicron BA.4/5), Moderna/Spikevax or Moderna/Spikevax Bivalent (Original/Omicron BA.1) or Nuvaxovid. For analysis the reference point for determining homologous and heterologous vaccination regimen may include the primary vaccine series or first booster.

2. HYPOTHESIS AND OBJECTIVES

2.1. Hypothesis

We hypothesise that one or more heterologous prime-boost-boost COVID-19 vaccine combinations will produce humoral and cellular immunity that is non-inferior to an homologous prime-boost-boost vaccination against wildtype SARS-CoV-2 and/or 1≥ VOC.

In Phases A-C of the study the primary 2 dose mRNA vaccine series was defined as 'Prime-boost'. For phase D we will define these 2 doses as 'Prime' and the 3rd vaccine dose as 'Boost'.

2.2. Summary of objective and outcome measures

Objectives	Outcome measures	Timepoint (s)
**Primary objective**		
Phase A-C: To determine whether heterologous prime-boost-boost COVID-19 vaccine regimens lead to non-inferior humoral immunity compared with homologous prime-boost-boost vaccine regimen against wildtype SARS-CoV-2 and/or 1≥ VOC<br><br>Phase D: To compare humoral immunity post-second booster against wildtype SARS-CoV-2 and/or 1≥ VOC	Level of SARS-CoV-2 anti-spike immunoglobulins	Day 28
**Secondary objectives**		

Phase A-C: To determine whether heterologous prime-boost-boost COVID-19 vaccine regimens lead to non-inferior humoral and cellular immunity compared with homologous prime-boost-boost vaccine regimen against wildtype SARS-CoV-2 and/or 1≥ VOC<br><br>Phase D: To compare humoral and cellular immunity post-second booster against wildtype SARS-CoV-2 and/or 1≥ VOC	Level of SARS-CoV-2 anti-spike immunoglobulins<br><br>Level of SARS-CoV-2 neutralising antibodies<br><br>Quantitative T-cell responses to spike proteins	Day 1, 7, 180, 360<br><br>Day 1, 7, 28, 180, 360<br><br>Day 1, 7, 28, 180, 360
Phase A-D: To assess the reactogenicity and safety of heterologous and homologous prime-boost-boost COVID-19 vaccine schedules	Solicited local and systemic reaction<br><br>Changes from baseline in laboratory safety measures (Only for Phase A-C)<br><br>Unsolicited adverse events (AEs)<br><br>Serious adverse events (SAEs), AEs of special interest (e.g. myocarditis, pericarditis), medically attended AEs	7 days after injection<br><br>7 days after injection<br><br>28 days after injection<br><br>Throughout the study
**Exploratory objectives**		
To determine whether vaccine efficacy differs between heterologous and homologous prime-boost-boost regimens	PCR-confirmed COVID-19 infections as recognised by Ministry of Health, Singapore<br><br>Positive for COVID-19 on self-administered Antigen Rapid Test (ART)/supervised self-administered ART/medical professional administered ART, in accordance with the Ministry of Health recommendations and guidelines	Throughout the study
To characterise and compare the mucosal immune response in nasal swab and saliva following COVID-19 vaccination with heterologous and homologous prime-boost-boost regimens	Level of SARS-CoV-2 anti-spike immunoglobulins	Day 1, 7, 28, 180, 360

To determine whether the administered mRNA vaccine (Phase A control group and intervention group 1 only) can be detected in the blood at ~1 week post vaccination*	qRT-PCR using primers targeting the sequence of the mRNA for SARS-CoV-2 spike protein	Day 7

**Residual leftover mRNA vaccine (that will otherwise be discarded) will be shared with laboratory collaborator (Prof Jerry Chan's team) for priming/ calibration purpose for the qRT-PCR assay*

2.3. Potential Risks and benefits:

a. End Points – Efficacy

There is the potential benefit to study participants in either control or experimental groups that the prime-boost-boost vaccine combination they receive will provide enhanced long-term humoral and cellular immunity (compared with not receiving a booster) against wildtype SARS-CoV-2 and/or 1≥ VOC.

For the wider community, it is expected that the information gained from this study may contribute to the development of a versatile, safe and effective vaccination programme against COVID-19.

b. End Points – Safety

The COVID-19 vaccines to be used in this study have proven efficacy and safety. It is possible that study participants in the experimental groups may be less well protected against SARS-CoV-2 infection compared with the control group, however, all participants are expected to receive a significant boost to protective immunity.

Similarly, based on extensive studies conducted overseas, no additional safety risks with the prime-boost-boost combinations are expected, including antibody-dependent enhancement or immunopathology as these were not observed as part of the clinical trials of the primary vaccine series. Safety of the prime-boost-boost vaccinations will be assessed by monitoring the incidence of SAEs and AEs. See product inserts for specific risks from individual vaccines.

Of the study procedures, phlebotomy may be associated with localised bruising and discomfort at the site of venepuncture. Infrequently fainting may occur. These will not be documented as AEs as may occur with any venepunctures and are not related to study products. The total volume of blood drawn over a 12-month period is less than 200mL, within limits recommended by DSRB for otherwise healthy volunteer adults.

3. STUDY POPULATION

3.1. List the number of subjects to be enrolled.

The maximum feasible target will be 100 participants per intervention group of the prime-boost-boost COVID-19 vaccination. A total of up to 600 participants was planned at the outset of this study, and 150 were enrolled across Phases A to C.

For Phase D we will enrol 250 individuals (see section 6). The recruitment period has been extending to 1 year and the follow-up period is 1 year.

3.2. Criteria for Recruitment

For phases A-C the key eligibility criterion was individuals who had received a homologous primary vaccines series with BNT162b2 or mRNA-1273 at least six months prior. For Phase D, the key eligibility criteria will be receipt of an mRNA booster vaccine at least five months prior.

3.3. Inclusion Criteria

1. Willing and able to provide informed consent for participation in this study;
2. Aged ≥21years at the time of study enrolment;
3. Received the second dose of BNT162b2 or mRNA-1273 Coronavirus Disease 2019 vaccines at least 6 months prior to enrolment;
4. Willing and able to comply with all scheduled visits, vaccination plan, laboratory tests and other study procedures.

For Phase D inclusion criteria 3 will be amended to 'Received the first booster dose of BNT162b2 or mRNA-1273 Coronavirus Disease 2019 vaccine at least 5 months prior to enrolment'. Only individuals recommended to receive a second booster dose under MOH recommendations will be enrolled.

3.4. Exclusion Criteria

1. Known history of SARS-CoV-2 or SARS-CoV-1 infection;
2. Previously received an investigational coronavirus vaccine;
3. Previously received a SARS-CoV-2 monoclonal antibody;
4. Current or planned simultaneous participation in another interventional study;
5. A history of anaphylaxis, urticaria, or other significant adverse reaction requiring medical intervention after receipt of a COVID-19 vaccine, or otherwise have a contraindication to one of the available study vaccines per the approved label;
6. Individuals who are immunocompromised (e.g. active leukaemia or lymphoma, generalised malignancy, aplastic anaemia, solid organ transplant, bone marrow transplant, current radiation therapy congenital immunodeficiency, HIV/AIDS with CD4 lymphocyte count < 200 and patients on immunosuppressant medications);
7. Received systemic immunosuppressants or immune-modifying drugs for >14 days in total within 6 months prior to Screening (for corticosteroids >/= 20 milligram per day of prednisone equivalent). Topical tacrolimus is allowed if not used within 14 days prior to Day 1;
8. Individuals who are pregnant or breast feeding;
9. Chronic illness that, in the opinion of the study team, is at a stage where it might interfere with trial conduct or completion;
10. Deprived of freedom by an administrative or court order, or in an emergency setting, or hospitalised involuntarily;
11. Current alcohol abuse or drug addiction that might interfere with the ability to comply with trial procedures in the opinion of the study team;
12. Moderate or severe acute illness/infection (according to study team's judgement) on the day of vaccination, or febrile illness (temperature ≥ 37.5°C). A prospective participant should not be included in the study until the condition has resolved or the febrile event has subsided.

For Phase D of the trial exclusion criteria 1 and 4 will no longer apply: exclusion criteria 1 is no longer relevant as it not a consideration in MOH recommendations for COVID-19 booster vaccination, and exclusion criteria 4 because of the broad recommendations from MOH for keeping up to date with COVID-19 boosters.

3.5. Withdrawal Criteria

The participant may be withdrawn prematurely from the trial if he/she withdraws consent to participate in the study. In such instances, the withdrawal will be applicable to both data collection as well as vaccination.

Participants may voluntarily decline the booster vaccination, or they may be withdrawn by their attending physician and/or by the study investigator(s) on the basis of the participant's best interest. Even if the participant did not receive the booster vaccination, collected samples will still be analysed up until the point the participant withdraws consent.

All participants, including those who declined the booster vaccination, will be followed up until 12 months, unless the subject withdraws informed consent or is lost to follow-up or have died.

3.6. Subject Replacement

Participants who drop out will not be replaced.

4. TRIAL SCHEDULE

The schedule of assessments and investigations is shown in Table 1:

**Trial activity**	**Screening**[1] **Day -28 to 0**	**Visit 1 Day 0**	**Visit 2 Day 7 (±2 days)**	**Visit 3 Day 28 (±7 days)**	**Visit 4 Day 180 (±14 days)**	**Final visit Day 360 (±14 days)**
**Enrolment**						
Informed consent[2]	X					
Eligibility	X					
Demographics	X					
Medical history	X					
**Study intervention**						
Confirmation of eligibility		X				
Randomisation[3]	X					
Vaccination[4]		X				
**Study procedures**						
Physical examination[5]	X	X	X	X		
Vital signs[6]	X	X	X	X	X	X
Pregnancy test[7]		X				
Medical review	X	X	X	X		
COVID-19 infection review[8]		X	X	X	X	X
AE evaluation[9]			X	X		
SAEs, AEs of special interest (e.g. myocarditis,		→	→	→	→	→

pericarditis), medically attended AEs						
**Safety laboratory (Phase A-C only)[10]**						
Haematology[11]	X		X			
Liver and renal panel[12]	X		X			
Cardiology panel[13]	X		X			
**Research laboratory**						
Blood samples for immunogenic analysis[14]	X	X[15]	X	X	X	X

Footnotes:

[1] Screening visit may be performed on the same day as Visit 1

[2] Informed consent must be signed prior to initiating any study procedures;

[3] Randomisation is subjected to vaccine availability;

[4] Visit 1: third vaccine shot for participant (first and only vaccine administered for the study);

[5] Physical examination will be done by a qualified study team member or a treating licensed healthcare provider. The physical examination on visit 1 will be conducted during screening prior to vaccination;

[6] Vital signs include pulse, systolic and diastolic blood pressure, respiratory rate and body temperature prior to vaccination and blood collection;

[7] For women of child bearing potential a urine pregnancy test will be conducted for Phases A-C of the study, and not for Phase D;

[8] At each study visit, a review for PCR-confirmed COVID-19 infection will be conducted. In most cases, such information will be available in the participant's HealthHub app. In addition, a review for any positive COVID-19 detected by self-administered Antigen Rapid Test (ART)/supervised self-administered ART/medical professional administered ART will be conducted. For positive results, the category of ART (whether it was self-administered or supervised or administered by a medical professional), the brand of the ART kit used (only if self-administered), and the date of ART positive will be captured;

[9] All AEs including a list of solicited and other events will be recorded for 7 days after vaccination (see diary version 3.0). Unsolicited AEs will be recorded up to day 28 after vaccination. SAE will be recorded from first vaccination until the end of study period at 12 months. They will be assessed via the listed study procedures, safety laboratory tests, and participant self-recorded diary;

[10] Safety laboratory bloods will only be monitored during Phases A-C of the study, and not for Phase D

[11] Haematology tests include full blood count with differential and platelet counts;

[12] Liver panel includes Albumin, total bilirubin, alkaline phosphatase (ALP), alanine transaminase (ALT); Renal panel includes sodium, potassium and creatinine;

[13] Cardiology panel include creatine kinase, troponin;

*[14] Blood samples will be collected at NCID research clinic, de-identified/coded before dispatch to local research laboratories such as A*STAR Singapore Immunology Network* and *NCID's National Public Health Laboratory. The study team based at NCID research clinic will maintain the codes linking the blood samples to its donor. Subjected to participant consent, any de-identified leftover blood samples may also be analysed for exploratory research to find new scientific information about coronaviruses and related diseases, which may occur locally or overseas;*

[15] Blood sample for immunogenic studies may be taken at Visit 1 instead of Screening if the blood draw is not possible for one reason or another. The study team will make every effort to complete all blood-taking in one seating to avoid pricking the participants more than once.

5. STUDY DESIGN

5.1. Summary of Study Design

PRIBIVAC is an adaptive, randomised, subject-blinded, controlled trial to assess the immunogenicity and safety of heterologous boost COVID-19 vaccination (intervention groups) compared with a homologous boost regimen (control group). For phases A-C of the study potential participants would have already received the second dose of a homologous prime-boost vaccination with BNT162b2 or mRNA-1273 at least six months prior to enrolment. For Phase D a booster dose of either BNT162b2 or mRNA-1273 would have been administered at least five months prior to enrolment.

In Phase A-C of the study the booster vaccine for the control arm will be the homologous mRNA vaccine (e.g. BNT162b2 + BNT162b2 + BNT162b2 or mRNA-1273 + mRNA-1273 + mRNA-1273), while for individuals randomised to intervention group 1 the mRNA booster vaccine administered will be heterologous to the primary series (e.g. BNT162b2 + BNT162b2 + mRNA-1273 or mRNA-1273 + mRNA-1273 + BNT162b2). The booster vaccine candidates for intervention groups 2 and 3 were an alternative COVID-19 vaccine.

Control group: Homologous mRNA booster vaccine
Intervention group 1: Heterologous mRNA booster vaccine
Intervention group 2: Non-mRNA booster vaccine A (i.e.COVAXIN®)
Intervention group 3: Non-mRNA booster vaccine B (i.e. Nuvaxovid)

Phase D of the study will examine the immune response following a second COVID-19 vaccine booster. Study participant will be randomised to receive one of three vaccines in equal proportions: Comirnaty or Comirnaty Bivalent (Original/Omicron BA.4/5), Moderna/Spikevax or Moderna/Spikevax Bivalent (Original/Omicron BA.1) or Nuvaxovid. Vaccines are obtained from the Singapore Ministry of Health stockpile. Where possible the bivalent Comirnaty/Spikevax vaccines will be administered to study participants, however, because of uncertainties in availability the monovalent may be administered if bivalent stock is not available in Singapore. For analysis the reference point for determining homologus and heterologous vaccination regimen may include the primary vaccine series or first booster.

COVID-19 vaccines used in the study may have been granted full or interim authorisation (under the Pandemic Special Access Route, PSAR) by the Health Sciences Authority (HSA), have been authorised via the Special Access Route (SAR) for vaccines as part of the Emergency Use List (EUL)[42] of the World Health Organization (WHO), or be under clinical development. BNT162b2, mRNA-1273 were authorised in Singapore under the PSAR, and on 3 Feb, HSA granted interim authorisation for Novavax's vaccine-manufactured under the name Nuvaxovid under PSAR. Nuvaxovid became the first non-mRNA vaccine accepted as an alternative for a boost vaccination in Singapore. Comirnaty Bivalent (Original/Omicron BA.4/5) and Moderna/Spikevax Bivalent (Original/Omicron BA.1) were authorised in Singapore under the PSAR on 11 Oct 2022 and 14 Sept 2022 respectively for use as a booster vaccine.

The non-mRNA vaccine COVAXIN® (Bharat Biotech) was approved by the World Health Organization (WHO) under the Emergency Use Listing (EUL) procedure on 3 Nov 2021 for use in two doses for primary vaccination and is available in Singapore via the SAR. The booster is not approved in the EUL.

Participants will be enrolled at the NCID research clinic. Participants will be blinded to the vaccine allocation (study team will not be blinded) to reduce the risk of bias in self-reported AEs. Participants will receive the blinded vaccination on study day 0 / visit 1. Participants will be observed for 30 minutes in the clinic after vaccination as per standard clinical practice. Participants will be monitored and followed up for AEs at Day 28 and SAEs throughout the study by physical examinations, laboratory tests and self-reporting. Blood samples will also be taken during screening or day 0, and at the follow-up visits at days 7, 28, 180, 360 post-enrolment for evaluation of immunogenicity and/or safety. Any missed follow-up visits by participants will not be considered a protocol deviation as it is beyond the control of the study team if a participant does not turn up for a follow-up visit, especially in a large-scale study.

For Phase A-C because of limited available data and the potential for inclusion of vaccines which were not approved as a booster, safety and futility was reviewed by an independent data and safety monitoring board (DSMB) after the first 10 participants in each of the intervention arms had completed assessments at study day 28. The DSMB was in a position to recommend discontinuation of a study arm on the basis of safety concerns or futility in immunogenicity. An outline of this safety monitoring plan is provided in Section 9.4 and more details will be provided in the DSMB charter.

For Phase D of the study we will study only COVID-19 vaccines approved for use by HSA. As such a DSMB will not be convened.

6. METHODS AND ASSESSMENTS

6.1. Randomisation and Blinding

Randomisation will be performed using a web-based randomisation system hosted by Singapore Clinical Research Institute (SCRI), in which a randomisation list with randomised permuted blocks will be generated by the trial statistician. Password secured accounts will be assigned to the site personnel who are responsible for the randomisation, where he/she can log into the randomisation system using the Internet.

For the first booster part of the trial randomisation will be stratified by the following criteria:

1. Age (<60, ≥60)
2. Primary vaccine series (BNT162b2 or mRNA-1273)
3. Time from 2nd vaccine dose administered (6-9 months, >9 months)

For the second booster part of the trial randomisation will be stratified by the following criteria:

1. Age (<60, ≥60)
2. Primary vaccine series (BNT162b2 or mRNA-1273)
3. First booster vaccine received (BNT162b2 or mRNA-1273)

Eligible participants will be randomised in equal proportions to each of the study arms that are open for randomisation. Enrolment to a study arm will be discontinued if recommended by DSMB or if the target sample size of 100 in the study arm has been reached. In the scenario where recruitment is discontinued in any of the intervention arms from 1 to 4 at the interim analysis, randomisation to the control arm may continue beyond 100 participants until the target sample size of 100 participants has been reached in the remaining intervention arms.

Allocation concealment will be maintained until the registration and randomisation process have been completed. For Phase A-D of the study, vaccine allocation will be single-blind, i.e. only participants will be blinded. Blinding will be maintained for the participants till their Day-28 Visits (Visit 3).

6.2. Contraception and Pregnancy Testing

For safety reasons, female participants of child-bearing potential will be asked to perform a urine pregnancy test for Phases A-C of the study, and not for Phase D – see trial schedule at section 4 for more information. A negative test result is required to be eligible for the study. At enrolment, all female participants of child-bearing potential will be reminded to use contraception or abstain from sexual intercourse until Visit 3 (Day 28).

6.3. Study Visits and Procedures

a. Screening Visit and Procedures

Participants will be screened based on the inclusion and exclusion criteria stated in Sections 3.3. and 3.4. If participants agree to join the study, they will be asked to sign the informed consent form (ICF) and provide information on relevant medical history. Randomisation will take place at this visit. In Phase A-C of the study blood will be taken from participants for the following clinical labs:

- Full blood count inclusive of differential blood count and platelet count;
- Liver Panel: Albumin, total bilirubin, ALP, ALT;
- Renal Panel: Sodium, potassium, creatinine;
- Cardiology Panel: Creatine kinase, troponin;

The results of the above clinical tests are for comparison with repeat tests at Day 7. Results will not affect eligibility to proceed with randomisation and study vaccination and hence will not be waited for prior to proceeding with Visit 1. These bloods will not be collected at Phase D given the established safety of the vaccines in use in this study as both primary vaccines series and boosters.

Assessment of the participant can occur on the same day as Visit 1, and must be carried out within 28 days prior to the start of the vaccination regimen. The screening visit may occur after five months have elapsed from the second dose of the primary vaccine series. However, the vaccination visit will be within six months per study eligibility criteria.

At the same time, bloods will be collected for research investigations as follows at either screening visit or visit 1:

Antibody response assays

To determine the presence and levels of anti-SARS-COV-2 in human sera, a standard ELISA will be used with the whole spike-protein, RBD fragment, or peptides encompassing dominant epitopes of the S and N proteins immobilised in the microplate wells. To identify the repertoire of epitopes induced by the vaccines, a peptide library ELISA will be used. This will provide fine resolution of the antibody responses against

the S-proteins at different time points and help to determine whether there is a fixation of the antibody response to certain epitopes (antigenic sin).

To examine the neutralising capacity of the antibodies in the human sera, two different assays will be performed: the sVNT and the pseudovirus or live-virus assay inhibition.

T-cell response assays

Quantitative T-cell responses to the vaccines will be measured using SARS-CoV-2 peptides from spike protein to stimulate the PBMCs isolated from donor's blood. To detect CD8 T-cells, PBMCs will be stimulated with two different peptide pools consisting of MHC-I peptides 8–10 amino acids in length. Negative controls using media and DMSO (~0.5%) will be used as reference. Assays will be performed in duplicates. The cell and peptides will be incubated in ELISpot 96-well plates. After 24 hours, the plates will be washed and assayed for IFNγ. To detect CD4 Th1/2 cells, PBMCs will be stimulated with two different peptide pools consisting of MHC-II peptides that are 15 amino acids in length. After 24 hours, the plates will be washed and assayed for IFNγ (CD8, Th1) or IL4 (Th2). Spot forming units (SFU) will be measured using IRIS reader (MabTech). Since these assays are done routinely in A*STAR laboratories and the epitopes have been identified, we do not foresee experimental roadblocks.

b. Study Visits and Procedures

- Visit 1 (Day 0, 0-28 days after screening)

During the first study visit, participants will undergo a physical examination (if Visit 1 does not occur on the same day as the Screening Visit). Results of clinical blood tests from the screening visit will be reviewed if applicable and available, and results of the urine pregnancy screen from women with child bearing potential. Urine pregnancy test will only be conducted for Phases A-C of the study, and not for Phase D. Eligibility to proceed with study will be confirmed by a study investigator. Research blood would be taken at this visit if it is not performed during screening.

After review the participant will be administered the vaccine and observed for the next 30 min as per clinical practice. The participant will be given a diary to record all the local and general symptoms experienced after receiving the vaccination. Local symptoms may include pain, redness, swelling, bruising, itchiness, muscle ache or movement limitation at the injection site. General symptoms may include headache, fever, nausea, vomiting, sore throat, cough, runny nose, tiredness and red (sore) eyes, as well as symptoms suggestive of COVID-19 infections (e.g. fever, cough and shortness of breath). This diary will cover 7 days post-vaccination and must be returned to the study team when the participant comes for the next visit.

- Visit 2 (Day 7, ±2 days)

After physical examination and medical review, blood will be taken from the participant for the laboratory (research and if applicable safety) tests as shown in the trial schedule. The study team will examine the participant's, record vital signs and record any AE or SAEs.

- Visit 3 (Day 28, ±7 days)

  After physical examination and medical review, blood will be taken from the participant for the laboratory research tests as shown in the trial schedule. The study team will examine the participant's, record vital signs and record any or SAEs. The study participant will be unblinded and vaccination dose recorded in subject's record.

- Visit 4 (Day 180, ±14 days)

  The subject will report to the clinic for monitoring of vital signs and recording of any SAEs. Blood will be taken from the participant for the laboratory research tests as shown in the trial schedule.

- Final Study Visit (Day 360, ±14 days)

  The participant will report to the clinic for monitoring of vital signs and recording of any SAEs. Blood will be taken from the participant for the laboratory research tests as shown in the trial schedule. Unless there are any SAEs that need to be followed up closely, the participant will be discharged from the study after this visit.

c. Post Study Follow up and Procedures

Unless there are any SAEs that need to be followed up closely, no post study follow-up and procedures need to be performed after the final study visit.

d. Discontinuation Visit and Procedures

Participants are free to withdraw from the study at any time without any consequence. Participants should be listed as having withdrawn consent only when they no longer wish to participate in the study and no longer authorise the study team to continue to obtain their outcome data.

Participants who withdraw from the study or are lost to follow-up after signing the ICF will not be replaced. The reason for participant withdrawal from the study will be recorded on the appropriate CRF.

7. TRIAL MATERIALS

7.1. Trial Product (s)

At the time of writing (02 Nov 2022), five COVID-19 vaccines (the monovalent mRNA vaccines BNT162b2 and mRNA-1273, the bivalent Comirnaty (Original/Omicron BA.4/5) and Moderna/Spikevax (Original/Omicron BA.1) vaccines, and the non-mRNA Nuvaxovid) have been authorised by HSA for use in Singapore. WHO approved Covaxin under the EUL procedure for use in two doses for primary vaccination. The booster is not approved in the EUL.

7.2. Storage and Drug Accountability

At the time of writing (02 Nov 2022), five COVID-19 vaccines (the monovalent mRNA vaccines BNT162b2 and mRNA-1273, the bivalent Comirnaty (Original/Omicron BA.4/5) and Moderna/Spikevax (Original/Omicron BA.1) vaccines, and the non-mRNA Nuvaxovid) have been authorised by HSA for use in Singapore. The storage condition, dosing, formulation and administration of the vaccines used in this study will be according to prevailing recommendations provided by MOH and the conditions of the PSAR approval. This study is following the MOH's guideline of giving BNT162b2 at IM 30 mcg (0.3 mL) per dose, mRNA-1273 at IM 50 mcg (0.25 mL) per dose, non-mRNA Nuvaxovid at IM 5mcg (0.5mL) per dose, Comirnaty Bivalent (Original/Omicron BA.4/5) at IM 30 mcg (0.3 mL) per dose, and Moderna/Spikevax Bivalent (Original/Omicron BA.1) at IM 50 mcg (0.5 mL) per dose. The booster dose is meant as a single dose.

WHO approved Covaxin under the EUL procedure for use in two doses for primary vaccination. The storage condition, dosing, formulation and administration of the vaccines used in this study will be according to the package insert approved by WHO EUL. As Covaxin has not been approved for booster, this study will be investigating Covaxin as a booster dose at single IM 6ug (0.5ml) per dose.

8. TREATMENT

8.1. Rationale for Selection of Dose

At the time of writing (02 Nov 2022), five COVID-19 vaccines (the monovalent mRNA vaccines BNT162b2 and mRNA-1273, the bivalent Comirnaty (Original/Omicron BA.4/5) and Moderna/Spikevax (Original/Omicron BA.1) vaccines, and the non-mRNA Nuvaxovid) have been authorised by HSA for use in Singapore. The storage condition, dosing, formulation and administration of the vaccines used in this study will be according to prevailing recommendations provided by MOH and the conditions of the PSAR approval. This study is following the MOH's guideline of giving BNT162b2 at IM 30 mcg (0.3 mL) per dose, mRNA-1273 at IM 50 mcg (0.25 mL) per dose, non-mRNA Nuvaxovid at IM 5mcg (0.5mL) per dose, Comirnaty Bivalent (Original/Omicron BA.4/5) at IM 30 mcg (0.3 mL) per dose, and Moderna/Spikevax Bivalent (Original/Omicron BA.1) at IM 50 mcg (0.5 mL) per dose. The booster dose is meant as a single dose.

WHO approved Covaxin under the EUL procedure for use in two doses for primary vaccination. The storage condition, dosing, formulation and administration of the vaccines used in this study will be according to the package insert approved by WHO EUL. As Covaxin has not been approved for booster, this study will be investigating Covaxin as a booster dose at single IM 6ug (0.5ml) per dose.

8.2. Study Drug Formulations

At the time of writing (02 Nov 2022), five COVID-19 vaccines (the monovalent mRNA vaccines BNT162b2 and mRNA-1273, the bivalent Comirnaty (Original/Omicron BA.4/5) and Moderna/Spikevax (Original/Omicron BA.1) vaccines, and the non-mRNA Nuvaxovid) have been authorised by HSA for use in Singapore. The storage condition, dosing, formulation and administration of the vaccines used in this study will be according to prevailing recommendations provided by MOH and the conditions of the PSAR approval. This study is

following the MOH's guideline of giving BNT162b2 at IM 30 mcg (0.3 mL) per dose, mRNA-1273 at IM 50 mcg (0.25 mL) per dose, non-mRNA Nuvaxovid at IM 5mcg (0.5mL) per dose, Comirnaty Bivalent (Original/Omicron BA.4/5) at IM 30 mcg (0.3 mL) per dose, and Moderna/Spikevax Bivalent (Original/Omicron BA.1) at IM 50 mcg (0.5 mL) per dose. The booster dose is meant as a single dose.

WHO approved Covaxin under the EUL procedure for use in two doses for primary vaccination. The storage condition, dosing, formulation and administration of the vaccines used in this study will be according to the package insert approved by WHO EUL. As Covaxin has not been approved for booster, this study will be investigating Covaxin as a booster dose at single IM 6ug (0.5ml) per dose.

8.3. Study Drug Administration

At the time of writing (02 Nov 2022), five COVID-19 vaccines (the mRNA vaccines BNT162b2 and mRNA-1273, the bivalent Comirnaty (Original/Omicron BA.4/5) and Moderna/Spikevax (Original/Omicron BA.1) vaccines, and the non-mRNA Nuvaxovid) have been authorised by HSA for use in Singapore. The storage condition, dosing, formulation and administration of the vaccines used in this study will be according to prevailing recommendations provided by MOH and the conditions of the PSAR approval. This study is following the MOH's guideline of giving BNT162b2 at IM 30 mcg (0.3 mL) per dose, mRNA-1273 at IM 50 mcg (0.25 mL) per dose, non-mRNA Nuvaxovid at IM 5mcg (0.5mL) per dose, Comirnaty Bivalent (Original/Omicron BA.4/5) at IM 30 mcg (0.3 mL) per dose, and Moderna/Spikevax Bivalent (Original/Omicron BA.1) at IM 50 mcg (0.5 mL) per dose. The booster dose is meant as a single dose.

WHO approved Covaxin under the EUL procedure for use in two doses for primary vaccination. The storage condition, dosing, formulation and administration of the vaccines used in this study will be according to the package insert approved by WHO EUL. As Covaxin has not been approved for booster, this study will be investigating Covaxin as a booster dose at single IM 6ug (0.5ml) per dose.

8.4. Specific Restrictions / Requirements

There will be no restrictions on medications, herbs, vitamins and mineral supplements imposed on study participants.

8.5. Blinding

Only study participants will be blinded to the vaccine allocation. This is to reduce the risk of bias in participant-reported AEs. The study participant will be unblinded at the Day-28 visit (Visit 3) and the vaccination details recorded in the participant's national immunisation record.

8.6. Concomitant therapy

There will be no restrictions on diet, exercise or concomitant medication imposed on participants, except for the restriction of receiving any other COVID-19 vaccines outside of this study.

9. SAFETY MEASUREMENTS

9.1. Definitions

Safety will be assessed using the FDA Guidance Document (2007): Toxicity Grading Scale for Healthy Adult and Adolescent Volunteers Enrolled in Preventive Vaccine Clinical Trial (http://www.fda.gov/BiologicsBloodVaccines/GuidanceComplianceRegulatoryInformation/Guidances/Vaccines/ucm074786.htm). Participants will be monitored up to Visit 2 (Day 7) for the occurrence and nature of any AEs.

All AEs will be entered in the appropriate e-CRF (including seriousness, grade, severity, relationship to the IP and action taken) and in the source documents. The hospital laboratory will perform investigational tests as specified in the trial schedule, including full blood count (with differential blood count and platelet count), liver panel (total bilirubin, ALP, ALT), renal panel (sodium, potassium and creatinine), creatine kinase, troponin, and pregnancy test if indicated.

For out-of-range values, clinical laboratory reports must be reviewed by a physician within 24 hours of receipt. Out-of-range values will be evaluated as either clinically significant (CS) or not clinically significant (NS). By definition, a value flagged as "CS" must be entered on the AE page in the CRF. The test may be repeated at the Investigator's discretion. The Investigator may use his own judgment to determine whether the abnormal finding has sufficient reasons to immediately withdraw the participant from the study.

Any laboratory value that meets the definition of a SAE (see Section 9.3.) must be reported.

9.2. Collecting, Recording and Reporting of "Unanticipated Problems Involving Risk to Subjects or Others" – UPIRTSO events to the NHG Domain Specific Review Boards (DSRB)

UPIRTSO events refers to problems, in general, to include any incident, experience, or outcome (including AEs) that meets ALL of the following criteria:

1. **Unexpected**
   In terms of nature, severity or frequency of the problem as described in the study documentation (eg: Protocol, Consent documents etc).

2. **Related or possibly related to participation in the research**
   Possibly related means there is a reasonable possibility that the problem may have been caused by the procedures involved in the research; and

3. **Risk of harm**
   Suggests that the research places participants or others at a greater risk of harm (including physical, psychological, economic, or social harm) than was previously known or recognized.

Reporting Timeline for UPIRTSO Events to the NHG DSRB.

1. Urgent Reporting: All problems involving local deaths, whether related or not, should be reported immediately – within 24 hours after first knowledge by the NHG investigator.

2. Expedited Reporting: All other problems must be reported as soon as possible but not later than 7 calendar days after first knowledge by the NHG investigator.

9.3. Collecting, Recording and Reporting of Serious Adverse Events (SAEs) to the Health Science Authority (HSA)

All SAEs that are unexpected and related to the study drug must be reported to HSA.

"A serious adverse event or serious adverse drug reaction is any untoward medical occurrence at any dose that:

- Results in death.
- Is life-threatening (immediate risk of death).
- Requires inpatient hospitalization or prolongation of existing hospitalization.
- Results in persistent or significant disability/incapacity.
- Results in congenital anomaly/birth defect.
- Is a Medically important event.

Medical and scientific judgment should be exercised in determining whether an event is an important medical event. An important medical event may not be immediately life threatening and/or result in death or hospitalization. However, if it is determined that the event may jeopardize the subject and/or may require intervention to prevent one of the other AE outcomes, the important medical event should be reported as serious."

All SAEs that are unexpected and related to the study drug will be reported. The investigator is responsible for informing HSA no later than 15 calendar days after first knowledge that the case qualifies for expedited reporting. Follow-information will be actively sought and submitted as it becomes available. For fatal or life-threatening cases, HSA will be notified as soon as possible but no later than 7 calendar days after first knowledge that a case qualifies, followed by a complete report within 8 additional calendar days.

9.4. Safety Monitoring Plan

An independent DSMB will be established to monitor the study during Phase A-C of the study (first booster). The DSMB will evaluate the study design and protocol, and review the accumulative data of safety and immunogenicity, as well as subject enrolment, protocol deviations, and data quality. The DSMB will meet after 10 participants in each intervention group have completed assessments at study day 28. Since participant recruitment may start at different time points for intervention groups 1 to 4 depending on availability of the vaccines, separate DSMB meetings may be needed for data review.

A DSMB Charter will be drawn up, which describes the scope of data to be reviewed, memberships of DSMB, terms of reference, decision-making process, and timing and frequency of interim analyses (with specification of stopping guidelines if any). An interim analysis report will be prepared for each DSMB data review, which will include summary data on participant enrolment, data quality, demographic and baseline characteristics, protocol deviation, safety data, and immunogenicity data. Following review of interim data, DSMB may recommend discontinuation of participant enrolment to a study arm on the basis of safety concern and/or

futility in immunogenicity compared with the control arm. The DSMB may request additional data review if necessary.

For Phase D of the study involving vaccines which have been authorised for use in Singapore by the HSA, study safety will be monitored by the PI. This will include monitoring rates of COVID-19 infections and SAEs between study groups.

9.5. Complaint Handling

The contact information of the PI and DSRB will be provided to all study participants, which is available in the Participant Information and Informed Consent Sheet.

10. DATA ANALYSIS

10.1. Data Quality Assurance

Study monitors must be allowed to visit the study site periodically to assess the data quality and study integrity. Study monitors will review the study records on site to directly compare them with source documents, discuss the conduct of the study with the Investigator, and verify that the facilities remain acceptable. Such visits will be scheduled in advance to allow arrangements of diaries and personnel as appropriate. In addition, the study may be evaluated by government inspectors who must be allowed access to CRFs, source documents and other study files.

10.2. Data Entry and Storage

A clinical database will be developed and validated to collect all clinical and laboratory related information. The database will include information on demographics, medical history, vaccine arm allocation, vital signs, and laboratory investigation tests. The study team will manage the data and will conduct quality control of the data following their own standard operating procedures.

11. SAMPLE SIZE AND STATISTICAL METHODS

11.1. Determination of Sample Size

Based on the data from our ongoing COVID-19 vaccine immune-monitoring study (SCOPE), the mean level of SARS-CoV-2 anti-spike immunoglobulins was 84% (SD=15%) at 28 days after the second dose. This was determined using a SARS-CoV-2 surrogate virus neutralisation test (sVNT) that detects total immuno-dominant neutralising antibodies targeting the viral spike (S) protein receptor-binding domain in an isotype- and species-independent manner. We expect immunogenicity will be boosted back to the same level after the booster dose in the control arm. With an assumed immunogenicity level of 81% in an intervention arm and a non-inferiority margin of -10%, a sample size of 87 subjects per arm will ensure concluding non-inferiority of the intervention arm against the control arm with 80% power. The sample size is calculated at a one-sided 2.5% significance level and allows for a 15% dropouts. A sample size will offer power of 85.1% based on the above parameters.

Since recruitment of subjects to intervention arms may be activated in a staggered way depending on availability of the vaccines, and recruitment to the control arm will continue until the target

sample size of 100 is achieved in each intervention arm, we prepare to enrol a double sample size in the control arm for the purpose of study planning. This gives a total sample size of up to 600 subjects.

For Phase D of the study, the objective is to estimate the mean difference in humoral immunity (log10-transformed anti-S at day 28) between Pfizer vs Moderna, Pfizer vs Nuvaxovid and Moderna vs Nuvaxovid received as 2nd booster (three mean difference estimations). No hypothesis testing will be performed.

The standard deviation of day 28 log10-transformed anti-S titre from Phase A of the study was assumed to apply to responses to the second booster (note data from those aged 60 years and above per current MOH advisory for a second COVID-19 booster). Using confidence interval approach for sample size calculation, a sample size of 50 per arm (after allowing 5% dropout rate) was estimated to produce two-sided 95% confidence interval with distance from difference in means to limits that is equal to 0.13 when standard deviation of log10-transformed anti-S titre is assumed to be 0.32 for both arms that is being compared.

11.2. Statistical and Analytical Plans

a. General Considerations

The safety analysis set consists of all participants who received the study vaccine. The efficacy analysis set is defined as all participants who are assessable with respect to immunogenicity after receiving the study vaccine. The analysis will include all study arms, including those in which participant enrolment is discontinued following interim analysis. Separate set of analyses will be done for Phase A-C and Phase D.

b. Safety Analyses

Data of AEs, SAEs, Grade 3 and 4 clinical or laboratory AEs will be summarised by study arm with frequency and proportion of participants having the event, as well as the number of events. For SAEs and Grade 3 and 4 AEs, the proportion of participants with the events will be provided together with its 95% confidence interval (CI).

c. Efficacy Analyses

Graphical plots will be produced for the various immunity endpoints to depict the change of immunity levels over time. Levels of SARS-CoV-2 anti-spike immunoglobulins will be summarized by study arm and by randomisation stratification factors. Mean difference and its 95% CI in levels of SARS-CoV-2 anti-spike immunoglobulins between an intervention arm and the control arm (for Phase A-C), and between pairwise arms (for Phase D) will be estimated from a general linear regression model which adjusts for level of SARS-CoV-2 anti-spike immunoglobulins at baseline, and randomisation stratification factors. If the lower bound of the 95% CI of Phase A-C SARS-CoV-2 anti-spike immunoglobulins falls above -10% (the pre-specified non-inferiority margin), non-inferiority of the intervention arm will be concluded. For Phase D, reported mean differences and 95% CIs are exploratory in nature. Analysis of repeated measurements of immunity endpoints will be performed with the use of mixed effects models that adjust for baseline values and randomisation stratification factors. Log-transformation of the data may be applied as needed before the analyses.

d. Describe the types of statistical interim analyses and stopping guidelines (if any) that are proposed, including their timing.

For Phase A-C of PRIBIVAC interim analyses will be performed for DSMB review after 10 participants from each of the intervention arm have completed assessments at study day 28.

DSMB may recommend discontinuation of participant enrolment to a study arm if any of the following criteria is met:
- Proportion of participants with SAE is at least 25% greater (in absolute difference) in the intervention arm compared with the control arm
- Proportion of participants with Grade 3 and 4 AEs is at least 25% greater (in absolute difference) in the intervention arm compared with the control arm
- Geometric mean ratio of anti-SARS-CoV-2 between the intervention arm and the control arm falls below 0.60.

The above guidelines may be revised in the DSMB charter, which implies the stopping guidelines will take precedence should there be any difference in the guidelines between the protocol and the DSMB charter.

For phase D of the study no formal stopping guidelines are provided, and no further data and safety review by DSMB will occur.

12. ETHICAL CONSIDERATIONS

12.1. Informed Consent

The PI is responsible for ensuring regulatory approval is obtained for the study.

The PI is also responsible for obtaining Institutional Review Board (IRB) approval for the protocol, and ensuring participant information sheet and ICF are in compliance with local regulatory requirements prior to enrolling any participant into the study. The approval letter/document must clearly identify the protocol and all documents approved by the IRB, including version number, date of the protocol, participant information sheet and ICF. The PI must also obtain approval for any amendments to the protocol or participant information sheet and ICF. The PI must comply with all IRB reporting requirements for all AEs, annual updates, end of study reports, and must agree to abide by any IRB conditions of approval. The PI (or designee) is responsible for ensuring freely-given consent is obtained from each potential participant prior to the conduct of any protocol-specific procedures. The PI may delegate the task of obtaining consent to appropriately qualified co-investigator(s). Consent must be documented by the participant's dated signature on the participant information sheet and ICF together with the dated signature of the person conducting the consent discussion.

If the participant is illiterate or a translator is required, an impartial witness should be present during the entire consent discussion. Once the discussion is complete, the participant must sign and date the ICF, if capable. The impartial witness must also sign and date the ICF along with the person who conducted the consent discussion.

A copy of the signed and dated participant information sheet and ICF must be given to the person prior to study participation. The participant must be informed in a timely manner of any new information that becomes available during the course of the study that may affect his/her willingness to continue study participation.

This study shall be conducted in accordance with the ethical principles laid out in the Declaration of Helsinki (most current issued version) and the National Statement on Ethical Conduct in Research Involving Humans (most current issued version).

12.2. IRB review

All relevant documents will be approved by the NHG DSRB before trial initiation in Singapore.

12.3. Confidentiality of Data and Patient Records

All study findings and documents will be regarded as confidential. The investigators and other study personnel must not disclose such information without prior written approval from the PI. Participant confidentiality will be strictly maintained to the extent possible under the law and local hospital policy. Identifiable information will be removed from any published data.

13. PUBLICATIONS

The data obtained from the study will be analysed as soon as possible after trial completion. Individual researchers will not publish data from the trial until the main study publication has been released. The study management committee will form the main writing committee.

14. RETENTION OF TRIAL DOCUMENTS

The PI will keep any records, study files or source documentation for a minimum of 15 years after the completion of the trial before being destroyed or erased. These documents may be retained for a longer period if required by the applicable regulatory requirements or institutional policy.

15. REFERENCES

1. Organization WH. *Novel Coronavirus – China.* 12 Jan 2020 2020.
2. Organization WH. *WHO Director-General's opening remarks at the media briefing on COVID-19 - 11 March 2020.* 11 Mar 2020 2020.
3. Tumban E. Lead SARS-CoV-2 Candidate Vaccines: Expectations from Phase III Trials and Recommendations Post-Vaccine Approval. *Viruses.* 2020;13(1).
4. *COVID-19 vaccine tracker.*
5. Polack FP, Thomas SJ, Kitchin N, et al. Safety and Efficacy of the BNT162b2 mRNA Covid-19 Vaccine. *New England Journal of Medicine.* 2020;383(27):2603-2615.
6. Baden LR, El Sahly HM, Essink B, et al. Efficacy and Safety of the mRNA-1273 SARS-CoV-2 Vaccine. *New England Journal of Medicine.* 2021;384(5):403-416.
7. Mallapaty S. China COVID vaccine reports mixed results — what does that mean for the pandemic? *Nature.* 2021.
8. Voysey M, Clemens SAC, Madhi SA, et al. Safety and efficacy of the ChAdOx1 nCoV-19 vaccine (AZD1222) against SARS-CoV-2: an interim analysis of four randomised controlled trials in Brazil, South Africa, and the UK. *The Lancet.* 2021;397(10269):99-111.
9. (CDC) CfDCaP. *The Advisory Committee on Immunization Practices' Interim Recommendation for Use of Janssen COVID-19 Vaccine — United States, February 2021.* 5 Mar 2021 2021.

10. Doria-Rose N, Suthar MS, Makowski M, et al. Antibody Persistence through 6 Months after the Second Dose of mRNA-1273 Vaccine for Covid-19. *The New England Journal of Medicine.* 2021.
11. Thomas SJ, Moreira ED, Kitchin N, et al. *Six Month Safety and Efficacy of the BNT162b2 mRNA COVID-19 Vaccine.* Infectious Diseases (except HIV/AIDS); 2021-07-28 2021.
12. *https://www.gov.il/BlobFolder/reports/vaccine-efficacy-safety-follow-up-committee/he/files_publications_corona_two-dose-vaccination-data.pdf.* 2021 2021.
13. Vogel G. *Do Delta 'breakthroughs' really mean vaccine protection is waning, and are boosters the answer?* 2021 2021.
14. (CDC) CfDCaP. *Science Brief: Emerging SARS-CoV-2 Variants.* 28 Jan 2021 2021.
15. Planas D, Veyer D, Baidaliuk A, et al. Reduced sensitivity of SARS-CoV-2 variant Delta to antibody neutralization. *Nature.* 2021;596(7871):276-280.
16. Chia WN, Zhu F, Ong SWX, et al. Dynamics of SARS-CoV-2 neutralising antibody responses and duration of immunity: a longitudinal study. *The Lancet Microbe.* 2021.
17. Callaway E. *COVID vaccine boosters: the most important questions.* 2021 2021.
18. Heller J, Lubell M. *Israel finds COVID-19 vaccine booster significantly lowers infection risk.* 2021 2021.
19. Mogus AT, Liu L, Jia M, et al. Virus-Like Particle Based Vaccines Elicit Neutralizing Antibodies against the HIV-1 Fusion Peptide. *Vaccines.* 2020;8(4).
20. Palgen J-L, Feraoun Y, Dzangué-Tchoupou G, et al. Optimize Prime/Boost Vaccine Strategies: Trained Immunity as a New Player in the Game. *Frontiers in Immunology.* 2021;12:612747.
21. He Q, Mao Q, An C, et al. Heterologous prime-boost: breaking the protective immune response bottleneck of COVID-19 vaccine candidates. *Emerging Microbes & Infections.* 2021;10(1):629-637.
22. Tan H-X, Juno JA, Lee WS, et al. Immunogenicity of prime-boost protein subunit vaccine strategies against SARS-CoV-2 in mice and macaques. *Nature Communications.* 2021;12(1):1403.
23. Milani A, Baesi K, Agi E, Marouf G, Ahmadi M, Bolhassani A. HIV-1 Accessory Proteins: Which one is Potentially Effective in Diagnosis and Vaccine Development? *Protein and Peptide Letters.* 2020.
24. Logunov DY, Dolzhikova IV, Shcheblyakov DV, et al. Safety and efficacy of an rAd26 and rAd5 vector-based heterologous prime-boost COVID-19 vaccine: an interim analysis of a randomised controlled phase 3 trial in Russia. *Lancet (London, England).* 2021;397(10275):671-681.
25. Logunov DY, Dolzhikova IV, Zubkova OV, et al. Safety and immunogenicity of an rAd26 and rAd5 vector-based heterologous prime-boost COVID-19 vaccine in two formulations: two open, non-randomised phase 1/2 studies from Russia. *The Lancet.* 2020;396(10255):887-897.
26. Cao Y, Yisimayi A, Jian F, et al. BA.2.12.1, BA.4 and BA.5 escape antibodies elicited by Omicron infection. *Nature.* 2022;608(7923):593-602.
27. Tuekprakhon A, Nutalai R, Dijokaite-Guraliuc A, et al. Antibody escape of SARS-CoV-2 Omicron BA.4 and BA.5 from vaccine and BA.1 serum. *Cell.* 2022;185(14):2422-2433 e2413.
28. Khan K, Karim F, Ganga Y, et al. Omicron BA.4/BA.5 escape neutralizing immunity elicited by BA.1 infection. *Nat Commun.* 2022;13(1):4686.
29. Wang Q, Guo Y, Iketani S, et al. Antibody evasion by SARS-CoV-2 Omicron subvariants BA.2.12.1, BA.4 and BA.5. *Nature.* 2022;608(7923):603-608.
30. Kimura I, Yamasoba D, Tamura T, et al. Virological characteristics of the SARS-CoV-2 Omicron BA.2 subvariants, including BA.4 and BA.5. *Cell.* 2022;185(21):3992-4007 e3916.
31. FDA. Coronavirus (COVID-19) Update: FDA Authorizes Moderna, Pfizer-BioNTech Bivalent COVID-19 Vaccines for Use as a Booster Dose. 2022; https://www.fda.gov/news-events/press-announcements/coronavirus-covid-19-update-fda-authorizes-moderna-pfizer-biontech-bivalent-covid-19-vaccines-use. Accessed 29 Oct 2022.
32. Scheaffer SM, Lee D, Whitener B, et al. Bivalent SARS-CoV-2 mRNA vaccines increase breadth of neutralization and protect against the BA.5 Omicron variant in mice. *Nat Med.* 2022.
33. Chalkias S, Harper C, Vrbicky K, et al. A Bivalent Omicron-Containing Booster Vaccine against Covid-19. *N Engl J Med.* 2022;387(14):1279-1291.

34. Munro APS, Janani L, Cornelius V, et al. Safety and immunogenicity of seven COVID-19 vaccines as a third dose (booster) following two doses of ChAdOx1 nCov-19 or BNT162b2 in the UK (COV-BOOST): a blinded, multicentre, randomised, controlled, phase 2 trial. *Lancet.* 2021;398(10318):2258-2276.
35. Andrews N, Stowe J, Kirsebom F, et al. Effectiveness of COVID-19 booster vaccines against COVID-19-related symptoms, hospitalization and death in England. *Nat Med.* 2022;28(4):831-837.
36. Regev-Yochay G, Gonen T, Gilboa M, et al. Efficacy of a Fourth Dose of Covid-19 mRNA Vaccine against Omicron. *N Engl J Med.* 2022;386(14):1377-1380.
37. Grikscheit K RH, Ghodatrian Z, et al. Characterization of antibody and T-cell response after second booster vaccination. *Research Square.* 2022.
38. Delbrueck Mea. Characterization of the humoral immune response to BNT162b2 in elderly residents of long-term care facilities five to seven months after vaccination. *medRxiv.* 2021.
39. Munro APS, Feng S, Janani L, et al. Safety, immunogenicity, and reactogenicity of BNT162b2 and mRNA-1273 COVID-19 vaccines given as fourth-dose boosters following two doses of ChAdOx1 nCoV-19 or BNT162b2 and a third dose of BNT162b2 (COV-BOOST): a multicentre, blinded, phase 2, randomised trial. *Lancet Infect Dis.* 2022.
40. Wang C, Horby PW, Hayden FG, Gao GF. A novel coronavirus outbreak of global health concern. *The Lancet.* 2020;395(10223):470-473.
41. Tan CW, Chia WN, Qin X, et al. A SARS-CoV-2 surrogate virus neutralization test based on antibody-mediated blockage of ACE2-spike protein-protein interaction. *Nature Biotechnology.* 2020;38(9):1073-1078.
42. *Status of COVID-19 Vaccines within WHO EUL/PQ evaluation process.* 2021 2021.
